# The *Toxoplasma* secreted effector *Tg*WIP modulates dendritic cell motility by activating host tyrosine phosphatases Shp1 and Shp2

**DOI:** 10.1007/s00018-024-05283-3

**Published:** 2024-07-09

**Authors:** Pavel Morales, Abbigale J. Brown, Lamba Omar Sangaré, Sheng Yang, Simon V.N.P. Kuihon, Baoyu Chen, Jeroen P.J. Saeij

**Affiliations:** 1 Department of Pathology, Microbiology and Immunology, School of Veterinary Medicine, University of California, Davis, Davis, CA USA; 2 Roy J. Carver Department of Biochemistry, Biophysics & Molecular Biology, Iowa State University, Ames, IA USA; 3 Present Address: Target & Protein Sciences, Johnson & Johnson, New Brunswick, USA

**Keywords:** *Toxoplasma*, *Tg*WIP, Dendritic cells, Shp1, Shp2, Dissemination

## Abstract

The obligate intracellular parasite *Toxoplasma gondii* causes life-threatening toxoplasmosis to immunocompromised individuals. The pathogenesis of *Toxoplasma* relies on its swift dissemination to the central nervous system through a ‘Trojan Horse’ mechanism using infected leukocytes as carriers. Previous work found *Tg*WIP, a protein secreted from *Toxoplasma*, played a role in altering the actin cytoskeleton and promoting cell migration in infected dendritic cells (DCs). However, the mechanism behind these changes was unknown. Here, we report that *Tg*WIP harbors two SH2-binding motifs that interact with tyrosine phosphatases Shp1 and Shp2, leading to phosphatase activation. DCs infected with *Toxoplasma* exhibited hypermigration, accompanying enhanced F-actin stress fibers and increased membrane protrusions such as filopodia and pseudopodia. By contrast, these phenotypes were abrogated in DCs infected with *Toxoplasma* expressing a mutant *Tg*WIP lacking the SH2-binding motifs. We further demonstrated that the Rho-associated kinase (Rock) is involved in the induction of these phenotypes, in a *Tg*WIP-Shp1/2 dependent manner. Collectively, the data uncover a molecular mechanism by which *Tg*WIP modulates the migration dynamics of infected DCs in vitro.

**Supplementary Information:**

The online version contains supplementary material available at 10.1007/s00018-024-05283-3.

## Introduction

*Toxoplasma gondii* is an obligate intracellular parasite that infects virtually all warm-blooded vertebrates, including humans and rodents, with an estimated one-third of the global population being chronically infected [1]. *Toxoplasma* dissemination from the point of entry in the intestinal tract plays a crucial role in the pathogenesis of toxoplasmosis. Upon reaching the intestinal tract, the parasite can cross into the small intestine and access the intestinal lamina propria, where it can then infect and replicate within a variety of cells, most notably leukocytes [2]. Subsequently, *Toxoplasma* can enter the blood circulation and rapidly spread into secondary lymphoid tissues and distal organs. For example, it can enter the eyes to cause ocular toxoplasmosis, or pass the blood-brain barrier (BBB) and invade the central nervous system (CNS) [3].

The rapid dissemination of *Toxoplasma* is hypothesized to be mediated by infected leukocytes, such as dendritic cells (DCs), where the parasite hijacks leukocytes and uses them as shuttles that carry parasites away from the site of infection and across biological barriers such as the BBB [4]. Aside from playing a central role in mediating essential immune responses after *Toxoplasma* infection [5], infected DCs play an important role in facilitating the parasite’s systemic dissemination [6, 7]. Previous studies have shown that *Toxoplasma*-infected DCs undergo cytoskeletal rearrangement changes that enhance their migratory properties, known as the hypermigratory phenotype [8]. Characteristics of the hypermigratory phenotype include dissolution of podosomes, which are adhesive actin-rich structures and sites of secretion of matrix metalloproteinases used to degrade the extracellular matrix (ECM) [9]. Additionally, infected DCs move at higher velocities in 2D and 3D matrigel environments and undergo enhanced transendothelial migration (TEM) across polarized endothelium [10]. Finally, the induced hypermigratory phenotype in infected DCs results in a more rapid in vivo dissemination of parasites to distant organs [6]. While some molecular signaling pathways in DCs, such as the non-canonical GABAergic signaling pathway [11], have been implicated in the hypermigratory phenotype, the exact molecular mechanisms by which *Toxoplasma* hijacks the motility of DCs to promote its own dissemination have yet to be determined.

We have previously identified a parasite rhoptry effector called the *Toxoplasma* Wave complex-Interacting Protein (*Tg*WIP). Secreted during host invasion, *Tg*WIP plays a key role in parasite dissemination to the brain, induction of the hypermigratory phenotype in infected DCs, and podosome dissolution [12]. However, the exact mechanisms of how *Tg*WIP induces these changes and promotes parasite dissemination have yet to be investigated. Here, we examined the role of *Tg*WIP in hijacking the migratory properties of DCs. We found that *Tg*WIP is phosphorylated by host kinases at two Src Homology 2 (SH2)-binding sequences. The phosphorylated tyrosines bind to the SH2 domains of host tyrosine phosphatases, Shp1 and Shp2, causing phosphatase activation. The interaction between *Tg*WIP and Shp1/2 contributes to the enhanced transmigration of infected DCs, which is accompanied by notable changes in the DC’s actin cytoskeleton, including cell spreading and the appearance of stress fibers. Additionally, we found that *Tg*WIP’s induction of F-actin stress fiber phenotype and enhanced transmigration is mediated by the Rho-associated kinase (Rock).

## Materials and methods

### Parasite culture

All the *Toxoplasma* parasite strains were routinely passaged in vitro in monolayers of HFF at 37 °C in 5% CO_2_ as previously described [13].

### Culture of cell lines

The THP-1 cell line was cultured in RPMI (complete media, CM), and the DC2.4 cell line in DMEM, 10% fetal bovine serum (FBS), 2mM L-glutamine, 10mM HEPES, 1X non-essential amino acids, 1mM sodium pyruvate, 100U/mL penicillin/streptomycin, 10 µg/mL gentamicin.

### THP-1 monocyte to DC differentiation

To induce differentiation into immature DCs, the human monocytic leukemia cell line THP-1 was seeded at 2 × 10^5^ cells/mL in a 75 mm adherent culture flask (in 20 mL of media) in the presence of human GM-CSF (100 ng/mL, Preprotech) and human IL-4 (100 ng/mL, Preprotech) for 7 days in CM. Fresh cytokine-supplemented medium was exchanged every 2 days. On day 7, the semi-adherent and cells in suspension were harvested, consisting of immature DCs (modified from Holken et al. [14]).

### Primary host cell culture

Bone marrow-derived Dendritic Cells (BMDCs) were isolated from 5 to 8 weeks old female CD1 mice (Charles River Laboratories) as previously described [11]. BMDCs were obtained by culturing murine bone marrow cells in RMPI 1640 with 10% FBS, 2mM L-glutamine, 10mM HEPES, 1X non-essential amino acids, 1mM sodium pyruvate, 100U/mL penicillin/streptomycin, 10 µg/mL gentamicin, referred to as complete medium (CM), and supplemented with recombinant mouse GM-CSF (40ng/mL, Peprotech) and mouse IL-4 (40ng/mL, Peprotech). Loosely adherent cells were harvested after 8 days of maturation. The medium was changed every 2 days in culture with fresh GM-CSF and IL-4 (modified from Inaba et al. [15]).

### Co-immunoprecipitation

DC2.4 cells or THP-1 derived DCs were grown in a 150 mm culture dish until 100% confluency and infected (MOI 3 to 5) for 4 h with ME49 *Tg*WIP^WT^ parasite expressing *Tg*WIP mutant strains or mock infected. Cells were then scraped in PBS, centrifuged and resuspended in 1 or 3 mL of lysis buffer (HEPES 10mM ph 7.9, MgCl2 1.5mM, KCl 10mM, EDTA 0.1mM, 5 mM dithiothereitol (DTT), 0.5mM, NP40 0.65%, cocktail of protease inhibitor (Roche), phenylmethylsulphonyl fluoride (PMSF) 0.5mM) for 30 min at 4 °C. The lysate was centrifuged for 30 min at 18,000 x g, 4 °C. Each sample was incubated with 35 µl of magnetic beads coupled with HA antibodies (Thermo scientific) and placed on a rotator overnight at 4 °C. The beads were washed three times with Tris-HCl 10mM pH7.5, NaCl 150mM, Triton-100 × 0.2%, PMSF 0.5mM, a cocktail of protease inhibitors (Roche), once more with Tris-HCl 62.5mM pH6.8 and beads were resuspended in 100 µl of this buffer.

### Immunoblotting

30 µl of the HA magnetic beads of each sample was used to run on a 12% SDS-PAGE. The proteins were transferred to a PVDF membrane, blocked 30 min with TBST, 5% nonfat dry milk. The membrane was blotted overnight at 4 °C with rat antibody against the HA tag (Roche, 1:500 dilution), phosphorylated Tyrosine (1:500 dilution), Shp1 (Invitrogen, 1:1000 dilution), or Shp2 (Invitrogen, 1:1000 dilution) antibodies, followed by their respective secondary antibodies. For Western blot analysis of phosphorylated Rock, 1 × 10^6^ murine BMDCs were seeded on tissue culture treated 6-well plates and infected with *Tg*WIP^WT^ or *Tg*WIP^Y150A/Y199A^*Toxoplasma* (MOI 7) for 4 h. Cells were then scraped in PBS, centrifuged and resuspended in 40 µl of RIPA lysis buffer (150 mM NaCl, 1% NP-40, 0.5% sodium deoxycholate, 0.1% SDS, 25mM Tris HCl pH 7.4, cocktail of protease inhibitor (Roche), 5 mM dithiothereitol (DTT)) for 30 min at 4 °C. The lysate was centrifuged for 30 min at 18,000 x g, 4 °C and run on 10% SDS-PAGE. The proteins were transferred to a PVDF membrane, blocked 30 min with TBST, 5% nonfat dry milk. The membrane was blotted overnight at 4 °C with rabbit polyclonal antibody against phospho-ROCK2 (Tyr722) (Invitrogen, 1:500 dilution), total ROCK2 (Cell Signaling, 1:1000), and mouse monoclonal SAG1 (clone CG52) followed by their respective secondary antibodies.

### Podosome assay

To test the podosome dissolution after *Toxoplasma* challenge, BMDCs were seeded on collagen coated glass coverslips overnight after which freshly egressed parasites were added at MOI 3 for 4 h, and the coverslips washed with PBS and fixed with 4% paraformaldehyde (PFA) for 20 min, permeabilized for 5 min with 0.1% Triton X-100, and blocked for 1 h with PBS with 3% (w/v) BSA. The coverslips were incubated with antibodies against SAG1 at room temperature for 1 h, washed and incubated with the respective fluorescent secondary antibodies, DAPI to stain the nucleus, and with Alexa Fluor 488 Phalloidin for 30 min. The coverslips were mounted with Vecta-Shield mounting oil and the microscopy was performed with NIS-Elements software (Nikon) and a digital camera (CoolSNAP EZ; Roper Scientific) connected to an inverted fluorescence microscope (eclipse Ti-S; Nikon) and either phase contrast or DIC imaging. Podosomes were identified and quantified as described in [8].

### DC gelatin degradation assay and image analysis

The in vitro gelatinolytic activity of DCs was analyzed by gelatinolysis of Oregon green 488 (OG 488)-conjugated porcine gelatin (Molecular probes). DCs (2.5 × 10^4^/well) infected with freshly egressed *Toxoplasma* tachyzoites (ME49-GFP, MOI as indicated) were deposited on OG-488 gelatin-coated glass coverslips and incubated for 24 h in CM. Cells were subsequently fixed (4% paraformaldehyde, Sigma), stained with DAPI (Invitrogen) or Alexa Fluor 594 Phalloidin (Thermo Fisher). Imaging and analysis were performed as indicated below. The in vitro gelatinolytic activity of DCs was analyzed by gelatinolysis of Cy-3-conjugated gelatin (molecular probes). After fixation (4% PFA, Sigma), cells were stained with DAPI (1:1000) and Alexa Fluor 594 Phalloidin (1:800). Using the software ImageJ version 1.54, the threshold of the green channel (Alexa Fluor 488 Phalloidin) was adjusted to distinguish single cells. The tool ‘‘Analyze particles’’ was used to count cells and measure the area of degradation. Gelatin degradation was defined as loss of signal (gelatin, Oregon green-488). The degradation of 100 cells was manually quantified for each condition. The “Analyze particles” tool was also used to analyze cell area and roundness of DCs using phalloidin to stain for F-actin and visualize the whole body of the cell; and used for nuclear area analysis using DAPI to visualize nucleus.

### Transmigration assay

The transmigration assays were performed by culturing BMDCs in CM and the addition of freshly egressed *Toxoplasma* strains (MOI 3) for 4 h. DCs were then transferred to transwell filters consisting of a porous membrane (8 μm; Corning) incubated at 37 °C. After 18 h, DCs from the bottom chamber were collected and quantified by hemacytometer. For transmigration assays using Rock inhibitor (Y-27,632, 40 µM), Src inhibitor (Dasatinib, 50nM), and Shp1/2 inhibitor (NSC-87,877, 100 µg), BMDCs were treated with inhibitors for 3 h before infection during the 4 h infection. Subsequently, inhibitor-treated BMDCs were spinned down and washed in media before being transferred to transwell filters.

### Flow cytometry analysis

Extracellular staining of BMDCs was performed using fluorescence activated cell sorting (FACS) buffer which was prepared by combining 7.4 pH PBS with 2% heat-inactivated fetal bovine serum (FBS). 2 × 10^5^ BMDCs were plated and either mock infected, LPS-treated, or infected with either wild-type or ∆*tgwip Toxoplasma* at MOI 1 for 18 h. After incubation, BMDCs were harvested, pelleted, and resuspended with FACS buffer. Cells were washed with FACS buffer one more time and subsequently incubated with Fc block using anti-mouse CD32/CD16 for 20 min on ice. Cells were then washed and incubated with CCR7 antibody for 30 min on ice. Cells were then washed with FACS buffer and fixed using 4% paraformaldehyde for 20 min. After fixing, cells were washed and resuspended in FACS buffer for analysis on the flow cytometer.

### Recombinant protein purification

To express recombinant *Tg*WIP from *E. coli*, a codon-optimized sequence was ordered as GeneArt Strings from Thermo Fisher and inserted into a modified pGEX expression vector (GE Healthcare) [16] using standard molecular biology procedures. The signal peptide comprising residues 2–33 was excluded and replaced with a Trp residue to facilitate protein concentration measurement using absorbance at 280 nm. Proteins were expressed using ArcticExpress™ (DE3) RIL cells (Agilent) in terrific broth following the manufacturer’s instructions. Briefly, cells were grown at 30 °C at 220 rpm till OD600 reached 1.5, when 0.5 mM IPTG was added to induce expression at 10 °C for 18–24 h. Harvested cells were resuspended in lysis buffer [20 mM Tris-HCl pH 8, 200 mM NaCl, 20% (w/v) glycerol, and 5 mM b-mercaptoethanol (BME)] and kept in -80 °C until use.

To purify *Tg*WIP, thawed cells were lysed on ice water by sonication and clarified by centrifugation at 19,500 rpm for 45 min at 4 °C. The supernatant was mixed with Glutathione Sepharose beads (Cytiva) for 30 min. After three washes using lysis buffer, the bound proteins were eluted in elution buffer [100 mM Tris-HCl pH 8.5, 30 mM reduced glutathione, and 20% (w/v) glycerol]. Eluted proteins were further purified by cation exchange chromatography using a 8-ml Source 15 S column [10 mM HEPES pH 7.0, 20% (w/v) glycerol, and 5 mM BME, with a gradient of 0–350 mM NaCl developed over 20 column volumes], followed by size exclusion chromatography using a 24-ml Superdex 200 Increase column equilibrated in 20 mM Tris-HCl pH 8, 100 mM NaCl, 20% (w/v) glycerol, and 1 mM DTT. To obtain untagged *Tg*WIP, proteins eluted from Glutathione Sepharose beads were treated with HRV 3 C protease overnight at room temperature to cleave off the GST tag. Treated samples were passed through an 8-ml Source 15Q column equilibrated in 10 mM Tris-HCl pH 8.5, 20% (w/v) glycerol, and 5 mM BME to absorb cleaved GST tag. Untagged *Tg*WIP was collected in the flow through and further purified by size exclusion chromatography using a 24-ml Superdex 75 Increase column equilibrated in 20 mM Tris-HCl pH 8, 100 mM NaCl, 20% (w/v) glycerol, and 1 mM DTT.

Human Shp1 (a.a. 1-595) was obtained as a codon-optimized GeneArt String from Thermo Fisher, and human Shp2 (a.a. 1-527) was a gift from Ben Neel (Addgene plasmid # 8322) [17]. Both Shp1 and Shp2 and their individual SH2 domains were inserted into a modified pMal expression vector (New England Biolabs) for expressing MBP-tagged proteins using ArcticExpress™ (DE3) RIL cells (Agilent) as described above for *Tg*WIP. Thawed cells were lysed on ice water by sonication and clarified by centrifugation at 19,500 rpm for 45 min at 4 °C. The supernatant was mixed with amylose beads (New England Biolabs) for 30 min. After 3 washes with lysis buffer, the bound protein was eluted in elution buffer [20 mM Tris-HCl pH 8, 2% (w/v) maltose, 20% (w/v) glycerol, 5 mM BME, and 1 mM MgCl_2_]. Eluted protein was then purified through anion exchange chromatography using an 8-ml Source 15Q column [10 mM Tris-HCl pH 8, 10% (w/v) glycerol, and 5 mM BME, and 1 mM MgCl_2_, with a gradient of 0–500 mM NaCl developed over 20 column volumes]. These proteins were then purified through a 24-ml Superdex 75 or Superdex 200 Increase column (Cytiva) equilibrated in gel filtration buffer [20 mM Tris-HCl pH 8, 20% (w/v) glycerol, 100 mM NaCl, 1 mM DTT, and 1 mM MgCl_2_].

The human Src kinase domain was purified following previous methods [18], using the expression vector from Amy Andreotti, Iowa State University. Briefly, Src was co-expressed with phosphatase YopH from a pCDFDuet vector using BL21 (DE3) T1R (Sigma). Cell pellets were thawed on ice water by sonication and clarified by centrifugation at 19,500 rpm for 45 min at 4 °C. Supernatant was mixed with Ni-NTA agarose resin (Qiagen) for 30 min. After 3 washes with lysis buffer, the bound protein was eluted in elution buffer [20 mM Tris-HCl pH 8, 500 mM NaCl, 500 mM imidazole pH 8, 5% (w/v) glycerol]. After dialysis overnight at 4 °C with two buffer changes in dialysis buffer [20 mM Tris-HCl pH 8, 100 mM NaCl, 5% (w/v) glycerol, 1 mM DTT], the kinase was purified using anion exchange chromatography using 4-ml Source 15Q column [10 mM Tris-HCl pH 8, 5% (w/v) glycerol, 5 mM BME] and then a Superdex 75 Increase column equilibrated in 20 mM Tris-HCl pH 8, 100 mM NaCl, 5% (w/v) glycerol, and 1 mM DTT.

All chromatography steps were performed using Cytiva columns on an ÄKTA Pure protein purification system. Purified *Tg*WIP, Shp1, Shp2, and Src were aliquoted in single-use volumes, flash frozen in liquid nitrogen, and stored in -80 °C for up to 1 year.

### In Vitro phosphorylation

Each reaction contained 20 nM Src kinase domain, 1 µM *Tg*WIP, 2 mM ATP, and 10 mM MgCl_2_ in 20 mM HEPES pH 7, 100 mM NaCl, 20% (w/v) glycerol, and 1 mM DTT. After overnight incubation at room temperature, the reactions were quenched using 20 mM EDTA. For negative controls, ATP/MgCl_2_ was replaced with 20 mM EDTA in the reaction. Phosphorylated samples were flash frozen in liquid nitrogen and kept in -80 °C till further analysis.

To validate and quantify the completeness of phosphorylation, a small portion of each reaction (20 µl) was analyzed by the Proteomics Core facility at University of Texas Southwestern Medical Center to determine intact protein molecular weight using electrospray ionization (ESI) quadrupole time-of-flight (QTOF) mass spectrometry. Phosphorylation typically reaches 90–100% completeness in our conditions. In parallel, phosphorylation was confirmed using Western blot. Briefly, samples in 10% SDS PAGE gel were transferred to a PVDF membrane using a Trans-Blot Turbo system (Bio-RAD). The membrane was blocked with 5% (w/v) BSA for one hour and then incubated with a 1:500 dilution of an HRP-conjugated antibody against phosphotyrosine (Cat#:sc-508 HRP, Santa Cruz) at 4 °C overnight. After three washes using Tris buffered saline (TBS)-Tween20, the membrane was developed using Amersham ECL Western Blotting Detection Reagent (Cat#: RPN2134, Cytiva) and a ChemiDoc XRS + system (Bio-RAD).

### Phosphatase activity assay

The impact of phosphorylated *Tg*WIP on both Shp1 and Shp2 activity was measured using a Shp2 activity assay kit (BPS bioscience, Cat# 79,330 [19]), following the manufacturer’s instructions. The kit measures the activity of full-length human Shp2 by monitoring the dephosphorylation of its substrate, Di-FMUP (6,8-Difluoro-4-Methylumbelliferyl Phosphate), and the spontaneous increase in fluorescence. Briefly, *Tg*WIP was incubated with full-length Shp2 (included in the kit) or Shp1 (a.a. 1-595, purified in-house) at room temperature for 1 h for Shp2 and 30 min for Shp1. Di-FMUP was then added to initiate the reaction. The final reaction contained 4 pg/µl Shp1 or Shp2 and 0.004 to 0.1 µM *Tg*WIP. Each reaction of 180 µl was divided into 3 separate wells in a 96-well flat-bottom black plate (Costar). Fluorescence intensity of each well was recorded every 18 s at 22 °C using a Spark plate reader (Tecan), with excitation at 360 nm and emission at 460 nm (15 nm bandwidth for both wavelengths). Typically, the reaction rate remains linear for at least 5 min. Shp1 and Shp2 activity in various conditions was normalized to the reaction rate of Shp1 and Shp2 without *Tg*WIP incubation.

### In Vitro GST pull down assay

GST pull-down experiments were performed as previously described [20]. Each reaction contained 100 pmol of GST-tagged *Tg*WIP, 200 pmol of MBP-tagged Shp1 or Shp2 (FL or individual SH2 domains), and 20 µl of Glutathione Sepharose beads (Cytiva) in 1 mL of binding buffer (100 mM HEPES pH 7, 100 mM NaCl, 20% (w/v) glycerol, and 5 mM BME). The reaction was mixed at 4 °C for 30 min. The beads were then washed three times with 1 mL of binding buffer. Bound proteins were eluted with GST elution buffer (10 mM Tris 8.5, 50 mM NaCl, 5% (w/v) glycerol, and 30 mM reduced glutathione) and further examined by SDS-PAGE.

## Results

### *Tg*WIP contains SH2-binding sites that could mediate interactions with Shp1 and Shp2

**Fig. 1:**
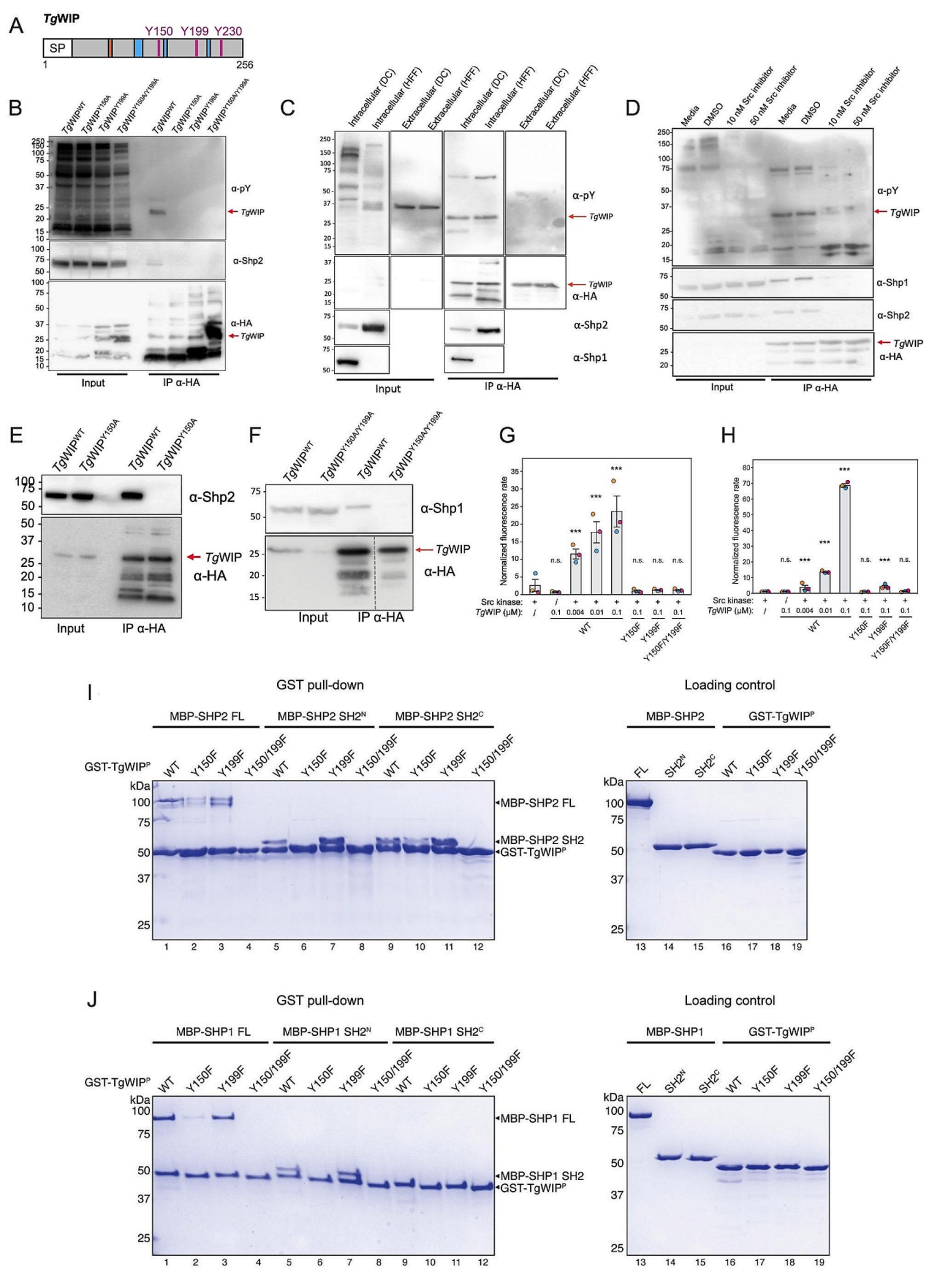
Phosphorylated *Tg*WIP binds to host cell tyrosine phosphatases Shp1 and Shp2. **A** Schematic showing the amino acid sequence of *Tg*WIP. Magenta color indicates the position of the three tyrosines in *Tg*WIP. Orange region represents the WRC interacting receptor sequence (WIRS) domain that could mediate *Tg*WIP’s interaction with the WAVE complex. Blue regions represent the proline rich regions (PRRs) that could mediate *Tg*WIP’s interactions with Nck and Grb2. **B** DCs were infected with parasites expressing wild-type *Tg*WIP (*Tg*WIP^WT^), or *Tg*WIP in which its SH2-binding motifs Y150 (*Tg*WIP^Y150A^), Y199 (*Tg*WIP^Y199A^), or both (*Tg*WIP^Y150A/Y199A^) are mutated to an alanine. Shown are the Western blots using phosphorylated tyrosine (pY) (1st membrane-probing antibody), HA (2nd membrane-probing antibody after stripping membrane), and Shp2 (3rd membrane-probing antibody after stripping membrane twice) antibodies on total lysate or immunoprecipitated *Tg*WIP. **C** The murine DC (DC2.4) and human foreskin fibroblast (HFF) cell lines were infected with type II ME49 *Δtgwip* complemented with HA-tagged wild-type *Tg*WIP (*Tg*WIP^WT^) for 4 h (intracellular) or until parasite egress (extracellular). Lysates were generated and *Tg*WIP immunoprecipitated. Shown are the Western blots using phosphorylated tyrosine (pY), HA, Shp1, and Shp2 antibodies, (probed in the same order as in C). **D** DC2.4 were treated with the Src kinase inhibitor Dasatinib at the indicated concentrations for 1 h and throughout infection with WT *Toxoplasma*. DMSO is the solvent control. Western blot analysis using antibodies against pY and HA. DCs were infected with *Tg*WIP^WT^ or with either **E ***Tg*WIP^Y150A^ or with **F ***Tg*WIP^Y150A/Y199A^*Toxoplasma.* Shown are the Western blots using antibodies against Shp2 in **E** and Shp1 in **F**. **G-H ** In vitro phosphatase assay of Shp2 in **G** Shp1 in **H** incubated with recombinant *Tg*WIP, with or without prior phosphorylation by recombinant Src kinase. Reaction rates, represented by linear fluorescence changes over time, were normalized against the rate of Shp2 or Shp1 without *Tg*WIP. Data from 3 biological repeats are indicated by different colors, with each data point showing the average from three technical repeats. Bar graph represents the mean and standard error of the 3 biological repeats. ANOVA with Tukey’s post hoc test was used. *n* = 3; *** for *p* < 0.0005 against the first column). n.s.: not significant. **I-J** Coomassie blue-stained SDS-PAGE gel showing GST-tagged, Src-phosphorylated *Tg*WIP (GST-TgWIP^P^) pulling down MBP-tagged full-length (FL) or individual SH2 domains of Shp2 in **I** or Shp1 in **J**. Loading controls contain 6 pmol of indicated prey proteins

Our previous work [12] established that upon cellular invasion, *Tg*WIP is secreted into the host cytosol, which leads to podosome dissolution and induces a hypermotility phenotype in the infected DCs. Our proteomic analysis of *Tg*WIP-binding proteins revealed that *Tg*WIP could interact with multiple host cytoplasmic proteins, including components of the pentameric WAVE regulatory complex (WRC), the adaptor proteins Nck1 and Grb2, and the tyrosine phosphatases Shp1 and Shp2. Among them, Shp1 and Shp2 are important regulators in both podosome formation and cell motility [21–24], and they share similar structures and regulation mechanisms. In the resting state, their central protein phosphatase domain is kept autoinhibited by two tandemly linked SH2 domains (reviewed in [25]). Activation is achieved by the binding of a phosphotyrosine (pY) peptide to the N-terminal SH2 domain, leading to its dissociation from the catalytic domain and the subsequent exposure of the phosphatase active site. The motifs identified by Shp1 and Shp2’s SH2 domains comprise a pY followed by specific amino acids. For Shp1, a nonpolar residue, such as alanine, following pY has been reported to be a preferential docking site for Shp1’s N-terminal SH2 domain [26]. For Shp2, the recognized consensus sequence is pY-X-X-pL with “X” representing any amino acid and “pL’’ signifying a hydrophobic amino acid, such as leucine or isoleucine [27]. Notably, *Tg*WIP contains three tyrosine residues, including Y150, Y199, and Y230 (Fig. 1A). Among them, both Y150 and Y199 are followed by an alanine-X-pL sequence (Ala-Glu-Leu and Ala-Thr-Leu, respectively) (Fig. S1). In contrast, Y230 is followed by Pro-His-His, which does not conform to the favored binding sequence for Shp1 or Shp2 [28]. This is also consistent with the predictions by both MoDPepInt and ScanSite servers [29, 30]. It is noteworthy that the Y-A-X-L motifs at Y150 and Y199 of *Tg*WIP are conserved even in the closely related non-*Toxoplasma* parasite, *Hammondia hammondi*, but Y230 is not (Fig. S1). Across nearly all examined *Toxoplasma* strains, the Y-X-X-L motifs of Y150 and Y199 are conserved, except the PRC2 strain, which contains Y199-X-X-P. These suggest the importance of Y150 and Y199 to *Tg*WIP’s function.

### *Tg*WIP selectively binds to and activates tyrosine phosphatases Shp1 and Shp2 via its phosphorylated tyrosine residues in infected DCs

To determine if *Tg*WIP’s tyrosine residues become phosphorylated, murine DCs (cell line DC2.4) were infected with *Toxoplasma* strains lacking *Tg*WIP (∆*tgwip*), or *∆tgwip* complemented with wild-type C-terminally HA tagged *Tg*WIP (*Tg*WIP^WT^) or its tyrosine-mutated variants (*Tg*WIP^Y150A^, *Tg*WIP^Y199A^, *Tg*WIP^Y150A/Y199A^) (Fig. 1B). Our immunofluorescence assays depicted *Tg*WIP as having a strong rhoptry organelle localization in both *Tg*WIP^WT^ and *Tg*WIP^Y150A/Y199A^ expressing *Toxoplasma* strains (Fig. S2A). Additionally, it is secreted into the host cytosol in comparable quantities (Fig. S2B/C). Subsequently, *Tg*WIP was immunoprecipitated using HA antibodies, and the tyrosine phosphorylation was evaluated by Western blot analysis using anti-phospho-tyrosine antibodies. The wild-type *Tg*WIP showed robust tyrosine phosphorylation, whereas none of its tyrosine-mutated variants showed phosphorylation (Fig. 1B), indicating that both Y150 and Y199 are necessary for efficient phosphorylation in cells.

To determine whether *Tg*WIP is phosphorylated after it is secreted into host cells or prior to secretion, we infected DC2.4 and Human Foreskin Fibroblasts (HFFs) with *Toxoplasma* expressing HA-tagged wild-type *Tg*WIP (Fig. 1C). We then immunoprecipitated *Tg*WIP from the cell lysate of infected cells versus from extracellular parasites egressed into the medium from the host cells. Our Western blot analysis shows that *Tg*WIP tyrosine phosphorylation was observed inside infected DCs and HFFs, and not in extracellular parasites, suggesting the involvement of a host kinase in mediating *Tg*WIP phosphorylation (Fig. 1C).

Given that *Tg*WIP undergoes phosphorylation within DCs and HFFs, we speculated that certain kinases in the host cell, likely of the Abl and/or Src family, may mediate this phosphorylation. To explore this possibility, we used the kinase inhibitor Dasatinib to block Abl or Src activity in infected DCs [31]. Remarkably, Western blot analysis showed an absence of *Tg*WIP phosphorylation in Dasatinib-treated DCs (Fig. 1D), suggesting a role for Abl or Src kinases in this process. Note that plaque assays showed no effect of Dasatinib on *Toxoplasma* invasion, aligning with *Tg*WIP phosphorylation occurring inside host cells (Fig. S3C**)**.

To investigate whether phosphorylated Y150 and Y199 of *Tg*WIP are required for its interaction with Shp1 and Shp2, we immunoprecipitated HA-tagged *Tg*WIP^WT^ and its tyrosine-mutated variant *Tg*WIP^Y150A/Y199A^ from infected murine DCs (Fig. 1E-F). Western blot analysis, using antibodies against Shp1 and Shp2, demonstrated binding to *Tg*WIP^WT^ but not to the tyrosine-mutated variant, indicating a requirement for these tyrosine residues in mediating the interactions (Fig. 1E-F). This analysis was also performed in infected HFFs and demonstrated *Tg*WIP^WT^ binding to Shp2 but not to Shp1 (Fig. 1C). However, the absence of Shp1 in the total lysate suggests that Shp1 is not strongly expressed in HFFs. Western blots conducted on human DCs derived from the THP-1 cell line, infected with either *Tg*WIP^WT^ or *Tg*WIP^Y150A/Y199A^, also show only *Tg*WIP^WT^ as tyrosine phosphorylated and directly binding to Shp2, but not *Tg*WIP^Y150A/Y199A^ (Fig. S3A).

To assess whether the observed interaction between phosphorylated *Tg*WIP and Shp1/2 is direct and whether the interaction is sufficient to activate the phosphatase, we purified recombinant *Tg*WIP and conducted an in vitro phosphatase activity assay. Prior to the assay, *Tg*WIP was phosphorylated in vitro using recombinant Src kinase domain. Notably, mass spectrometry and Western blot analyses of various *Tg*WIP tyrosine mutants revealed that Src efficiently phosphorylated both Y150 and Y199 to near completion, but did not phosphorylate Y230 at all (Fig. S4C). We then measured Shp2 phosphatase activity in the presence of either unphosphorylated *Tg*WIP or its phosphorylated form (indicated as Src + in Fig. 1G). Remarkably, phosphorylated WT *Tg*WIP significantly enhanced Shp2 activity in a dose-dependent manner, while unphosphorylated *Tg*WIP had no impact (Fig. 1G). It is worth noting that phosphorylation of a single tyrosine within *Tg*WIP, as in the Y150F or Y199F mutant, could not activate Shp2, mirroring the behavior of the double tyrosine mutant, suggesting that the two phospho-tyrosines act cooperatively to activate Shp2. Similar results were obtained for Shp1 (Fig. 1H).

To determine the specificity of phosphorylated Y150 and Y199 of *Tg*WIP for individual SH2 domains of Shp2, we conducted in vitro GST pull-down assays. We found that phosphorylated GST-tagged *Tg*WIP (GST-*Tg*WIP^P^), but not the tyrosine-mutated variant, Y150F/Y199F, specifically retained MBP-tagged Shp2 FL, N-terminal SH2 domain (MBP-SH2^N^), and C-terminal SH2 domain (MBP-SH2^C^) (Fig. 1I). This is consistent with the immunoprecipitation data showing phosphorylated *Tg*WIP interacted with Shp2 (Fig. 1B, E-F). Interestingly, although both tyrosines were crucial for activating Shp2 (Fig. 1G), Y150 seemed to play a dominant role in binding to FL Shp2 and both of its SH2 domains. The Y150F mutation abolished the binding to SH2^N^ and substantially reduced the binding to FL Shp2 and SH2^C^, while the Y199F mutation did not seem to affect the interactions (Fig. 1I). These data suggest that phosphorylated Y150 can bind to both SH2 domains with high affinity, while Y199 prefers to bind to SH2^C^, albeit with lower affinity. This is consistent with previous studies showing the sequence preference of individual SH2 domains of Shp2 [26, 32]. Despite the difference in binding affinity and preference, it is plausible that Y150 and Y199 need to work cooperatively to bind and activate Shp2, with phosphorylated Y150 likely binding to SH2^N^ first, which facilitates the binding of Y199 to SH2^C^, leading to the stabilization of the activated, open conformation of Shp2.

We similarly examined the interaction between *Tg*WIP and Shp1 using GST pull-down. The results revealed a similar binding specificity, in that Y150 plays a dominant role in binding to Shp1 **(**Fig. 1J). However, unlike the binding to Shp2, *Tg*WIP does not seem to have a detectable interaction with SH2^C^ in our experimental conditions **(**Fig. 1J). It remains to be determined whether both tyrosines are required for fully activating Shp1.

In conclusion, our findings indicate that upon parasite invasion into DCs, *Tg*WIP is phosphorylated at two tyrosine residues, Y150 and Y199, in both murine and human DCs, likely by host Abl and/or Src kinases. The phosphorylated *Tg*WIP directly interacts with Shp1 and Shp2, leading to phosphatase activation.

### *Tg*WIP’s interactions with Shp1/2 are not involved in podosome dissolution in infected DCs

**Fig. 2:**
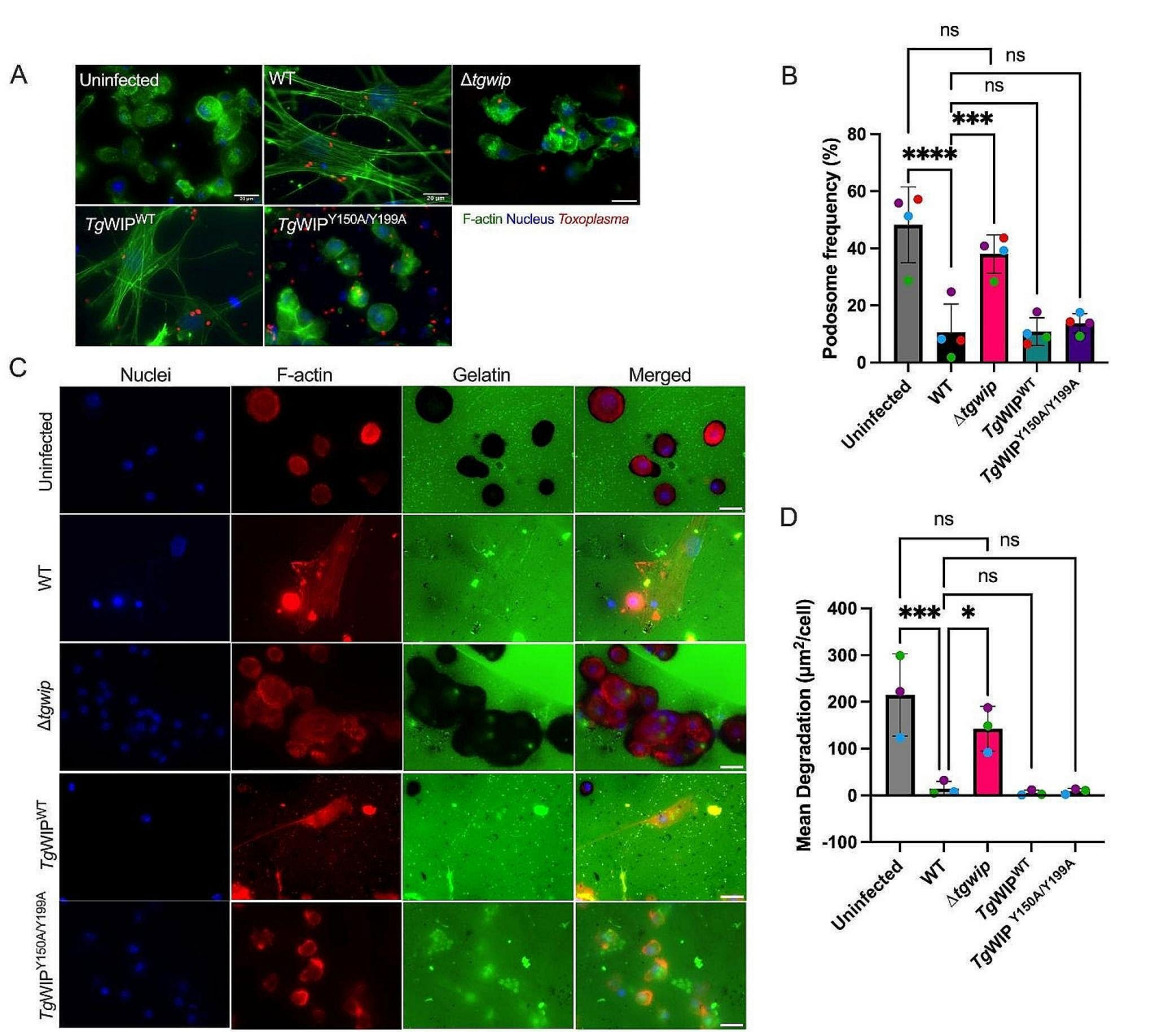
Podosome dissolution is independent of *Tg*WIP’s interactions with Shp1/2. **A** BMDCs were infected with wild-type (WT), *∆tgwip*, *Tg*WIP^WT^, or *Tg*WIP^Y150A/Y199A ^*Toxoplasma* strains for 4 h. The cell actin structure and podosomes were visualized with 488 Alexa Fluor Phalloidin, the parasite expressed RFP (WT) or GFP (mutants), and the nucleus with DAPI. **B** Quantification of the percentage of BMDCs containing podosomes 4 h post infection (p.i.). **C** Pericellular proteolysis of BMDCs upon challenge with *Toxoplasma* strains mentioned above. Representative immunofluorescence images of non-infected and infected BMDCs on fluorescent gelatin and phalloidin (F-actin), performed as detailed in Experimental Procedures. **D** Bar graph shows the mean area of gelatin degradation per Field Of View (FOV) related to the cell number (µm^2^/cell) for DCs that were infected with wild-type, *∆tgwip*, *Tg*WIP^WT^, or *Tg*WIP^Y150A/Y199A^*Toxoplasma* strains (*n* = 3). Data are from 3 independent experiments performed in duplicate. Asterisk (*), (***), (****) indicate significant differences, *p* < 0.05, < 0.0002, and < 0.0001, respectively; ns, non-significant difference; two-way ANOVA, Dunnett’s multiple comparisons test

Given the role of *Tg*WIP in inducing podosome dissolution in infected DCs, we sought to determine the impact of *Tg*WIP- Shp1/2 interactions on podosome formation. We assessed murine bone-marrow derived DCs (BMDCs) infected with wild-type, ∆*tgwip, Tg*WIP^WT^, and *Toxoplasma Tg*WIP^Y150A/Y199A^ using immunofluorescence assays of DCs stained for F-actin (Fig. 2A). In line with our previous experiments, the percent of podosome-containing DCs was significantly higher when they were infected with *Toxoplasma ∆tgwip* than with WT *Toxoplasma* (Fig. 2B).

However, DCs infected with *Toxoplasma Tg*WIP^Y150A/Y199A^ displayed podosome dissolution similar to those infected with WT *Toxoplasma*, suggesting that *Tg*WIP phosphorylation did not play a role in this process (Fig. 2B). We additionally assessed podosome dissolution in human THP-1 monocyte-derived DCs. Similarly, the podosome frequency in THP-1 DCs infected with *Toxoplasma* ∆*tgwip* was higher than with DCs infected with WT *Toxoplasma* (Fig. S3B). Notably, THP-1 DCs infected with *Toxoplasma* ∆*tgwip* had reduced podosomes compared to uninfected DCs, a finding not observed in murine BMDCs, suggesting other factors from *Toxoplasma* also contribute to podosome dissolution in THP-1 DCs. To further determine a possible role of *Tg*WIP-Shp1/2 interactions in podosome dysfunction, we quantified the matrix degradation of gelatin by DCs infected with WT or *Tg*WIP^Y150A/Y199A^ parasites (Fig. 2C, D). The proteolysis of *Toxoplasma Tg*WIP^Y150A/Y199A^ infected DCs showed no significant difference to the gelatin degradation of wild-type-infected DCs, which is consistent with our immunofluorescence assessment of podosome formation, indicating that *Tg*WIP phosphorylation or its interaction with Shp1/2 is not involved in the dissolution of podosomes in infected DCs.

### *Tg*WIP’s interactions with Shp1/2 leads to enhanced transmigration in infected DCs

**Fig. 3:**
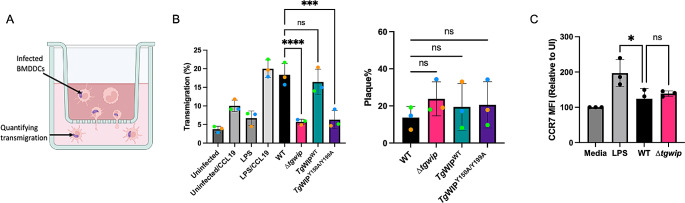
*Tg*WIP’s interactions with Shp1/2 lead to enhanced transmigration in infected BMDCs. **A** Schematic of experimental setup of transwell transmigration assays to assess BMDC motility. Uninfected BMDCs or BMDCs infected with wild-type, ∆*tgwip, Tg*WIP^WT^, or *Toxoplasma Tg*WIP^Y150A/Y199A^ were loaded into the upper chamber of the transwell. After 18 h incubation, the number of transmigrated BMDCs was quantified. As controls, uninfected BMDCs that were either treated with LPS for 24 h or left unstimulated were loaded into the upper chamber of the transwell with the presence or absence of the chemoattractant CCL19. **B** Transmigration frequencies (using empty transwell system) of unchallenged and *Toxoplasma*-challenged primary murine DCs related to total added cell numbers. Bar graphs show the average percentage of total transmigrated *Toxoplasma*-infected DCs, from 3 independent experiments performed in duplicate (*n* = 3). **C** Plaque assay of WT, ∆*tgwip, Tg*WIP^WT^, or *Tg*WIP^Y150A/Y199A^*Toxoplasma*. Bar graph shows the average percent of plaques counted at day 6. **D** Bar graphs represent the average mean fluorescent intensity (MFI) of CCR7 expression of wild-type, ∆*tgwip* infected, or LPS-treated BMDCs relative to uninfected BMDCs (media). Data are from 3 independent experiments performed in duplicate. Asterisks (****) indicate significant differences, p-value < 0.0001: ns, non-significant difference; two-way ANOVA, Dunnett’s multiple comparisons test

We then examined the role of *Tg*WIP-Shp1/2 interactions in inducing the hypermotility phenotype in infected DCs. An established feature of the hypermotility phenotype is the enhanced transmigration of infected DCs across endothelial layers and through empty transwells. Consistent with previous results, DCs infected with WT *Toxoplasma* had significantly enhanced transmigration frequencies compared to uninfected DCs, but had similar transmigration frequencies compared to that of uninfected DCs when they were stimulated with LPS and attracted to chemoattractant CCL19 in the media of the bottom chamber of the tranwell (Fig. 3B). The enhanced transmigration could not be observed when DCs were infected with ∆*tgwip* parasites, but was fully recovered in DCs infected with *∆tgwip* complemented with WT *Tg*WIP. DCs infected with *∆tgwip* parasites complemented with *Tg*WIP^Y150A/Y199A^ did not rescue this phenotype, showing transmigration similar to ∆*tgwip*-infected DCs, suggesting *Tg*WIP phosphorylation plays a key role in inducing transmigration in infected DCs (Fig. 3B, C).

The directed migration of DCs to secondary lymphoid organs is orchestrated via chemotactic cues, notably via the upregulation of the C-C chemokine receptor 7 (CCR7) [33]. Prior studies have reported an increased expression of CCR7 in DCs following 24 h infection with *Toxoplasma* [8]. To investigate whether *Tg*WIP modulates transmigration of infected DCs via upregulating CCR7, we performed flow cytometry analyses to compare CCR7 expression levels in DCs infected with either wild-type or ∆*tgwip Toxoplasma* strains. This analysis was conducted after 18 h of infection, which aligns with the timing of transmigration analysis. We saw no significant differences in CCR7 expression between these two groups (Fig. 3D). Given that the analysis of DC transmigration occurred after an 18 h incubation, these results indicate that deletion of *Tg*WIP does not modulate CCR7 post 18 h infection in vitro. Consequently, the enhanced transmigration conferred by *Tg*WIP-Shp1/2 interactions is likely not due to enhanced CCR7-mediated chemotaxis.

### *Tg*WIP’s interactions with Shp1/2 leads to actin cytoskeletal rearrangement

**Fig. 4:**
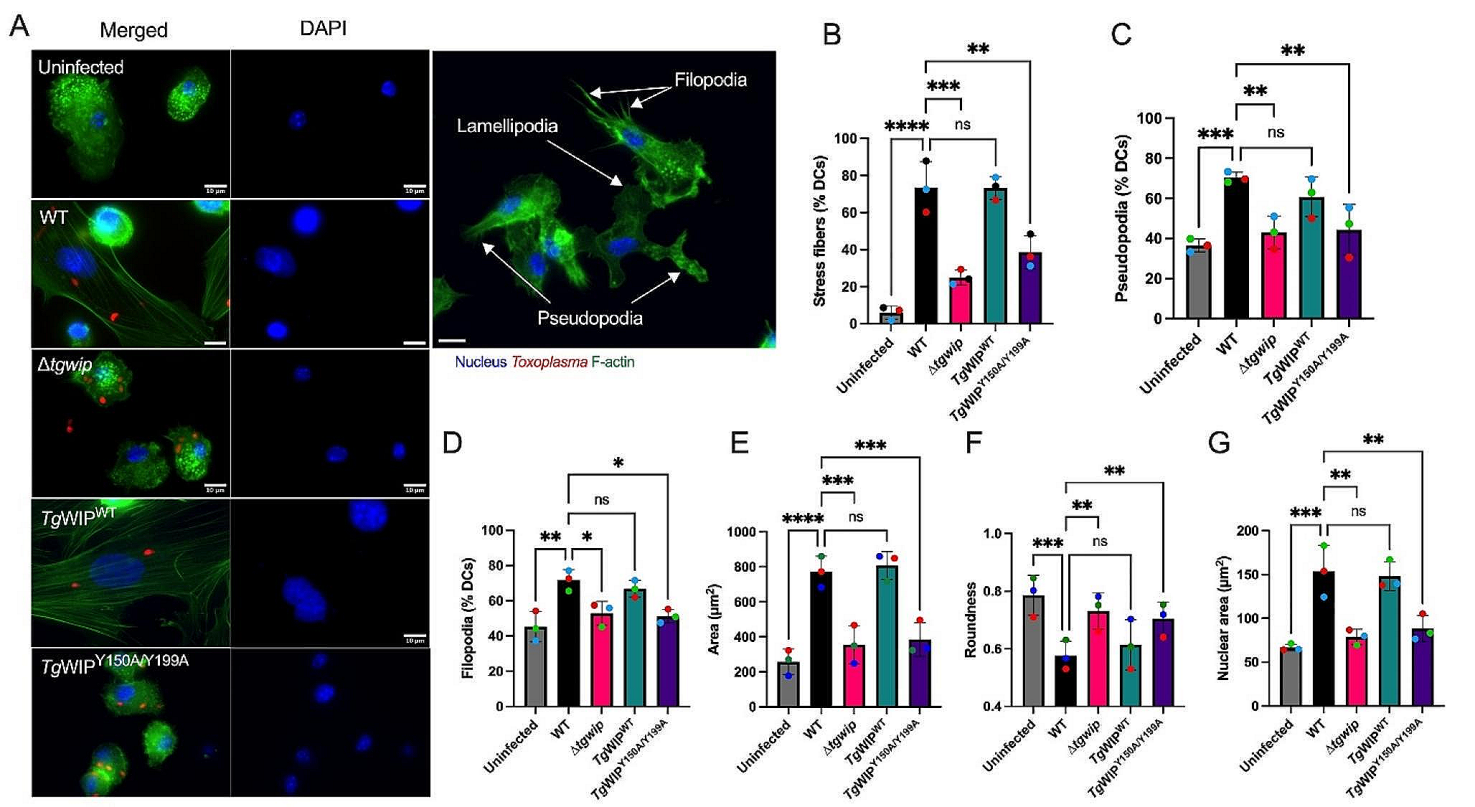
BMDCs infected with *Tg*WIP^Y150A/Y199A^*Toxoplasma* have reduced actin cytoskeleton modulation. **A)** BMDCs were infected with wild-type, *∆tgwip*, *Tg*WIP^WT^, or *Tg*WIP^Y150A/Y199A^*Toxoplasma* strains for 4 h. The BMDC actin structure was visualized with 488 Alexa Fluor Phalloidin, the parasite expressing RFP and the nucleus with DAPI. Quantification analysis representing the percent of uninfected BMDCs or BMDCs infected with the *Toxoplasma* strains mentioned above expressing. Arrows point to filopodia, lamellipodia, and pseudopodia. **B** F-actin stress fibers **C** pseudopodia **D** filopodia or quantification of **E** cell area, **F** cell roundness, and **G** nuclear area after 4 h p.i. **H** Immunofluorescence images of uninfected or infected BMDCs stained for F-actin and DAPI. Data are from at least 3 independent experiments performed in duplicate. Scale bar measures 10 μm. Asterisks (****) indicate significant differences, p-value < 0.0001: ns, non-significant difference; two-way ANOVA, Dunnett’s multiple comparisons test

The dynamic alterations in the actin cytoskeleton play an important role in DC migration, notably, in the formation of contractile structures such as stress fibers [34]. We and others have demonstrated that *Toxoplasma*-infected DCs undergo marked morphological changes, including the formation of stress fibers [8, 12]. Given that *Tg*WIP-Shp1/2 interactions enhance DC transmigration, we wanted to determine whether these interactions contributed to actin cytoskeletal rearrangements of infected DCs. For this, we used Immunofluorescence assays with F-actin staining to compare the morphology of the actin cytoskeleton of uninfected DCs with those infected by *Toxoplasma* (Fig. 4A). DCs infected with wild-type parasites not only displayed F-actin stress fibers (Fig. 4B), but also exhibited an increase in cell membrane protrusions, cell area, and a distinct loss of cellular roundness (Fig. 4C-G). Additionally, DCs infected with WT parasites exhibited an expanded nuclear area (Fig. 4A). In contrast, these morphological changes were absent in DCs infected with ∆*tgwip* parasites or those expressing *Tg*WIP^Y150A/Y199A^. Furthermore, incubation with supernatants from DCs, either uninfected or infected with either WT or ∆*tgwip* strains, did not induce the stress fiber phenotype in fresh DCs, suggesting that the effect of *Tg*WIP on host DC actin cytoskeleton was cell autonomous (Fig. S4A-C). Together, these data suggest that phosphorylation of *Tg*WIP and its subsequent interaction with host Shp1 and Shp2 play a key role in modulating the actin cytoskeleton of *Toxoplasma*-infected DCs.

### Src and Shp1/2 are important for the induction of the stress fiber phenotype in *Toxoplasma* infected DCs

**Fig. 5:**
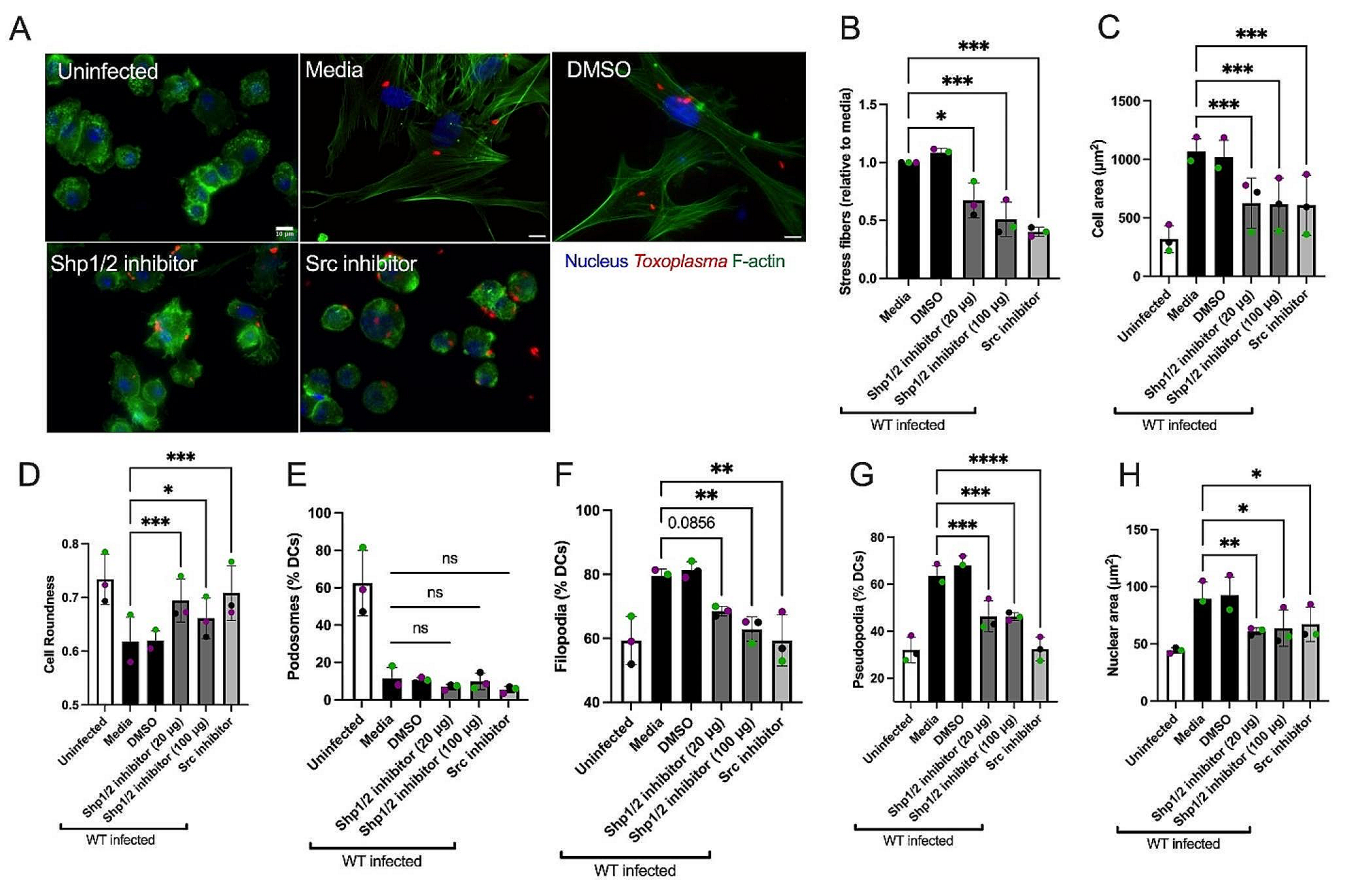
Impact of Src kinase and Shp1/2 inhibition on stress fiber formation in BMDCs infected with *Toxoplasma*. **A** BMDCs were pretreated with either 50nM Dasatinib for 3 h, or either 20 µg/mL or 100 µg/mL Shp1/2 inhibitor overnight, or DMSO solvent control overnight, or left in media, followed by infection with RFP + wild-type ME49 *Toxoplasma*. The BMDC actin structure was subsequently assessed using Alexa Fluor 488-conjugated Phalloidin. **B** The proportion of BMDCs demonstrating F-actin stress fiber formation relative to media. **C** Mean cell area. **D** Mean cell roundness. Percentage of BMDCs expressing **E** podosomes, **F** filopodia and **G** Pseudopodia. **H** Mean nuclear area. Data are from at least three independent experiments, each performed in duplicate. Quantification was performed on at least 100 uninfected BMDCs (media) or wild-type *Toxoplasma* infected BMDCs treated with corresponding inhibitors. Scale bar measures 10 μm. Asterisk (*), (**), (***), (****) indicate significant differences, p-value < 0.05, *p* < 0.005, *P* < 0.0002, p-value < 0.0001, respectively; ns, non-significant difference; two-way ANOVA, Dunnett’s multiple comparisons test

Given that the Abl/Src kinase inhibitor Dasatinib not only inhibited *Tg*WIP’s tyrosine phosphorylation in infected DCs, but also abrogated *Tg*WIP’s binding to both Shp1 and Shp2 (Fig. 1D), we assessed the role of Src kinases and Shp1/2 phosphatases in mediating stress fiber formation in *Toxoplasma* infected BMDCs. In addition to using Dasatinib to inhibit Abl/Src kinases, we used NSC-87,877 to specifically inhibit the catalytic activities of Shp1/2 and used immunofluorescence assays to determine how the inhibition of their activities affected the actin cytoskeleton of DCs infected with WT *Toxoplasma*. We found that upon treatment with either Dasatinib or NSC-87,877 infected DCs exhibited a reversal of the phenotypes induced by *Toxoplasma* infection. These included the loss of stress fiber formation (Fig. 5A-B), a decrease in cell and nuclear area (Fig. 5C, H), an increase of cell roundness (Fig. 5D), and reduced formation of both filopodia (Fig. 5F) and pseudopodia (Fig. 5G). In contrast, podosome dissolution was not reversed by the same treatment (Fig. 5E), consistent with our observation that podosome dissolution is independent of *Tg*WIP phosphorylation or its interactions with Shp1/2. Taken together, these data underscore the importance of Src-mediated tyrosine phosphorylation of *Tg*WIP and its subsequent interaction with Shp1/2 in modulating the actin cytoskeleton of *Toxoplasma* infected DCs.

### Rock is important for the induction of the stress fiber phenotype and enhanced cell motility in *Toxoplasma* infected DCs

**Fig. 6:**
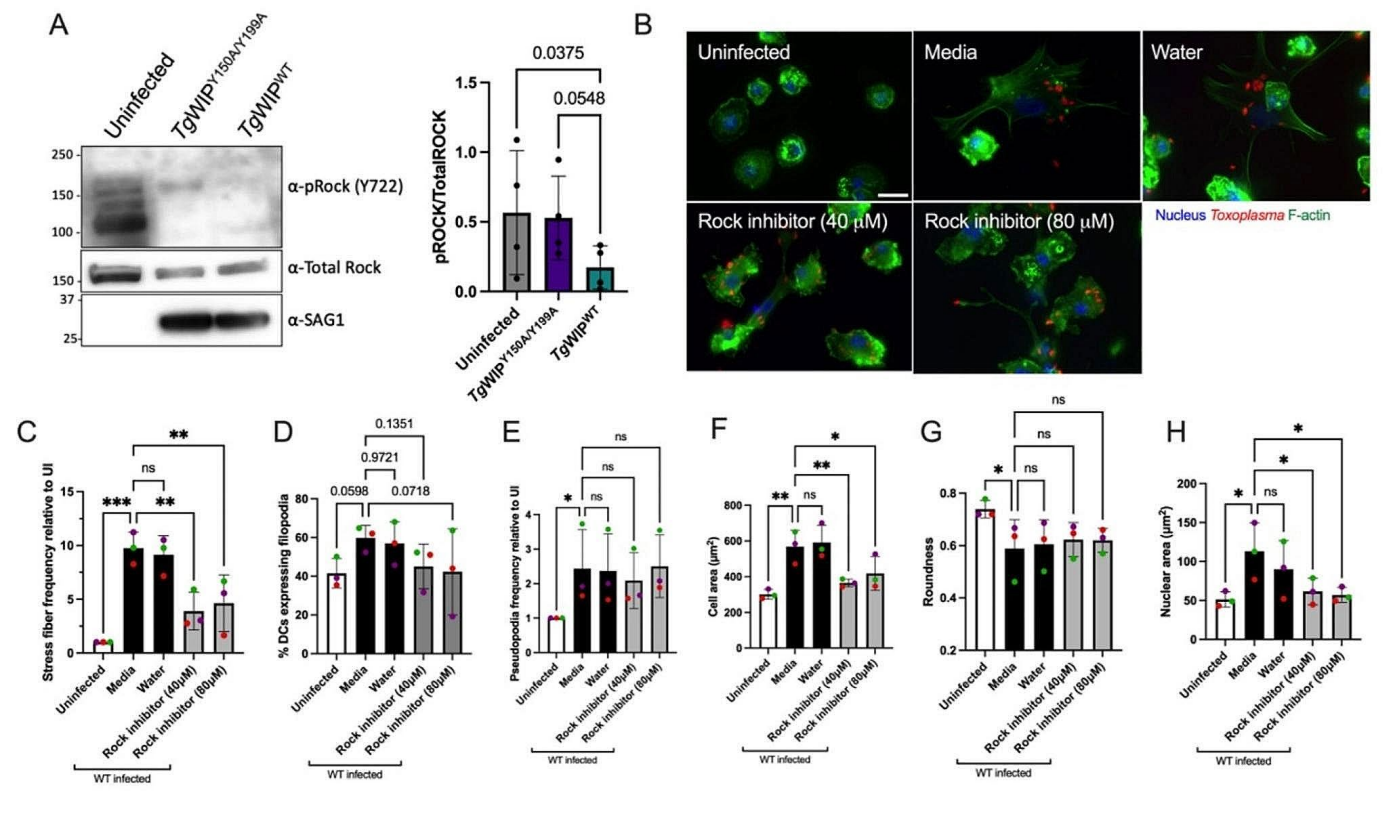
*Tg*WIP-Shp1/2 interactions lead to tyrosine dephosphorylation of the Rho-associated kinase (Rock) in infected BMDCs. **A** BMDCs were infected with either *Tg*WIP^WT^ or with *Tg*WIP^Y150A/Y199A ^*Toxoplasma* for 4 h at MOI 7. Shown are the Western blots against phosphorylated Rock at 722 tyrosine, total Rock protein, and SAG1. Bar graph shows the relative Rock Y722 phosphorylation quantified over the total Rock protein levels, in BMDCs infected with *Tg*WIP^WT^ compared to uninfected BMDCs and BMDCs infected with *Tg*WIP^Y150A/Y199A^. **B** BMDCs were pre-treated with either 40 µM or 80 µM Rock inhibitor (Y-27,632) for 3 h, and solvent control, or left uninfected. BMDCs were then infected with wild-type ME49 RFP + *Toxoplasma* and the BMDC actin structure was analyzed using 488 Alexa Fluor Phalloidin. Scale bar measures 20 μm. Quantification analysis representing the percent of uninfected BMDCs or wild-type *Toxoplasma* infected BMDCs treated with Rock inhibitor of **C** F-actin stress fibers relative to uninfected (UI), **D** filopodia, **E** pseudopodia, **F** mean cell area, **G** mean cell roundness and **H** mean nuclear area. Data are from at least 3 independent experiments performed in duplicate. Asterisk (*), (**), (***), (****) indicate significant differences, p-value < 0.05, *p* < 0.005, *P* < 0.0002, p-value < 0.0001, respectively; ns, non-significant difference; two-way ANOVA, Dunnett’s multiple comparisons test

**Fig. 7:**
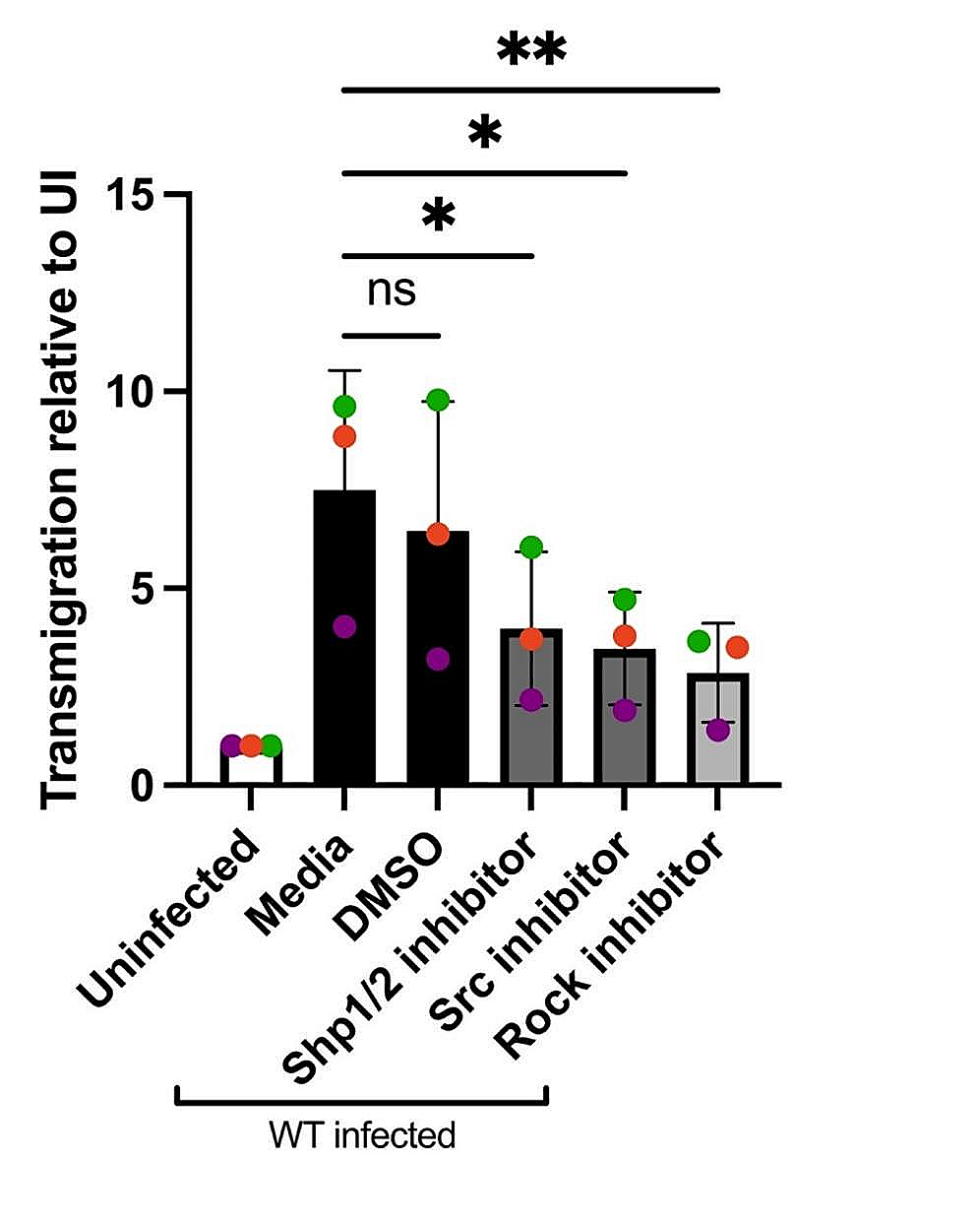
Wildtype-infected BMDCs treated with Src, Shp1/2, and Rock inhibitors have reduced transmigration frequencies. **A** Transmigration frequencies (using empty transwell system) of unchallenged and *Toxoplasma*-challenged BMDCs related to total added cell numbers. BMDCs were pre-treated with either Rock inhibitor (Y-27,632, 40 µM), Src inhibitor (Dasatinib, 50nM), Shp1/2 inhibitor (NSC-87,877, 100 µg), or solvent control (DMSO) for 3 h. BMDCs were then infected with wild-type *Toxoplasma* and loaded into the upper chamber of the transwell. After 18 h incubation, the number of transmigrated BMDCs was quantified. Bar graphs show the average percentage of total transmigrated *Toxoplasma*-infected DCs relative to uninfected DCs, from 3 independent experiments performed in duplicate (*n* = 3). Asterisk (*), (**), (***), (****) indicate significant differences, p-value < 0.05, *p* < 0.005, *P* < 0.0002, p-value < 0.0001, respectively; ns, non-significant difference; two-way ANOVA, Dunnett’s multiple comparisons test

The Rho-associated kinase (Rock) is a central protein involved in F-actin stress fiber formation, cell motility, and is a direct substrate of Shp2. Shp2 can dephosphorylate Rock Y722 thereby increasing the activity of Rock [23]. Therefore, we next sought to investigate whether the *Tg*WIP-mediated activation of Shp2 leads to the dephosphorylation of Rock’s Y722, leading to stress fiber formation and enhanced cell motility in infected DCs. Our Western blot analysis demonstrates lower phosphorylation levels of Rock’s Y722 in BMDCs infected *Tg*WIP^WT^ compared to BMDCs left uninfected or infected with *Tg*WIP^Y150A/Y199A ^*Toxoplasma* (Fig. 6A). Furthermore, the frequency of F-actin stress fiber expression in BMDCs infected with wild-type *Toxplasma* was significantly reduced when treated with the Rock inhibitor Y-27,632 (Fig. 6B-C). BMDC cell area and nuclear area were also reduced in infected BMDCs treated with Rock inhibitor, however, treatment with Rock inhibitor did not affect cell protrusions such as filopodia and pseudopodia, nor did it affect roundness in infected BMDCs (Fig. 6D-E, G). Furthermore, the inhibition of Src, Shp1/2, and Rock reduced the enhanced transmigration of wild-type infected BMDCs (Fig. 7A). There was no change in the infection rate of DCs treated with the Rock inhibitor (Fig. S6B). These data suggest that the *Tg*WIP-mediated activation of Shp2 leads to the activation of Rock and therefore inducing F-actin stress fibers and an enhanced transmigration of BMDCs infected with *Toxoplasma*.

## Discussion

The swift dissemination of *Toxoplasma* has been shown to be facilitated by infected leukocytes, particularly DCs, by a “Trojan-Horse” mechanism. Within minutes after active invasion by *Toxoplasma*, DCs undergo rapid morphological changes, coinciding with a hypermotility phenotype which heightens the cell’s ability to move and migrate across polarized endothelial cell monolayers [6]. Hypermigration of infected DCs has been linked to enhanced dissemination and higher parasite loads in mice after *Toxoplasma* infection. Furthermore, pretreatment of *Toxoplasma* with an inhibitor of rhoptry secretion and invasion, abrogated the hypermotility and morphological changes in DCs [8]. Collectively, this suggests that the active invasion process and the release of rhoptry proteins, is crucial for inducing morphological changes in DCs.

*Tg*WIP is a secreted rhoptry protein that is involved in modulating the actin cytoskeleton and motility of infected DCs, and is important for *Toxoplasma* dissemination. However, the mechanisms to how *Tg*WIP modulates the actin cytoskeleton and motility of infected DCs is unknown. In the present study, we demonstrated that *Tg*WIP modulates the actin cytoskeleton and motility of infected DCs via its interactions with Shp1 and Shp2 tyrosine phosphatases. Our data support a model that once secreted into the DC cytosol, *Tg*WIP is phosphorylated by Abl or Src kinases at Y150 and Y199. This phosphorylation allows *Tg*WIP to bind to SH2 domains of Shp1 and Shp2, which in turn stimulates their phosphatase activity. The activation of Shp1/2 leads to significant morphological changes in the host DCs, including the formation of F-actin stress fibers, cell spreading and polarization, and the formation of membrane protrusions such as pseudopodia and filopodia. Consequently, the parasitized DCs undergo a significantly enhanced transmigration across transwell filters in the absence of chemotactic stimuli, which is in line with previous studies that the *Toxoplasma* induced migration of DCs is not dependent of CCR7 [6]. Whether *Tg*WIP-Shp1/2 interactions also mediate *Toxoplasma* infected DC’s enhanced migration across endothelial monolayers has yet to be investigated.

Other pathogens hijack the activity of Shp1/2 phosphatases for microbial survival and pathogenesis. For example, the *Helicobacter pylori* virulence factor, cytotoxin-associated gene A (CagA) [35] is tyrosine phosphorylated by Src family kinases, upon which it binds to and activates the Shp2 phosphatase, conferring host cells with neoplastic traits and promoting gastric carcinogenesis [36]. Additionally, CagA expressing gastric epithelial cells undergo morphological changes, including an elongated, spindle-shaped morphology, and have increased expression of pseudopodia [37]. Furthermore, CagA-expressing cells acquired a migratory and invasive phenotype.

The actin structures that play a fundamental role in cell motility are actin filled 3D pseudopodia, filopodial bundles beneath the plasma membrane, and contractile actin stress fibers in the cytoplasm [38, 39]. Shp1 and Shp2 are tyrosine phosphatases well documented to be involved in the regulation of podosomes, F-actin stress fibers, and membrane protrusions [23, 40–42]. Stress fiber assembly is regulated by a signaling cascade involving the RhoA small GTPase [43]. RhoA binds to and activates the Rho-associated kinase (Rock), which promotes stress fiber formation by phosphorylating myosin light chains (MLCs) and inactivation of myosin light chain phosphatase (MLCP) to generate contraction force necessary for cell migration. The phosphorylation of MLC also regulates pseudopodia extension and retraction via RhoA signaling [44]. Additionally, the signaling pathways mediated by Rho-Rock impact the interactions between the actin cytoskeleton and integrins, which plays a role in cell shape and morphology. The kinase activity of Rock, in addition to being controlled by Rho-mediated mechanisms, is also regulated by phosphorylation at tyrosine 722 (Y722) [23]. Phosphorylation at Rock Y722 reduces its affinity for binding to GTP-bound Rho, thereby inhibiting the Rho-mediated activation of Rock.

Rock1 and Rock2 are isoforms which share 65% identity in their overall amino acid sequences [45]. Nonetheless, notable differences exist between these isoforms, particularly, in their regulatory mechanisms. In our study, we exclusively considered Rock2 due to several factors. Firstly, the understanding of the role of Y722 in regulating Rock1’s kinase activity, as well as the phosphatases targeting this site, remains limited compared to Rock2. Additionally, there are no publications detailing Rock1’s Y722 as a target of Shp1/2. Notably, Rock1’s involvement in stress fiber assembly and cell contraction is mainly regulated by the small GTPase RhoE via direct binding to Rock [46], which provides further importance to our focus on Rock2. Furthermore, the Rock inhibitor utilized in this study, Y-27,632, targets both Rock1 and Rock2 [47], thereby suggesting a potential role of Rock1 in *Tg*WIP-Shp1/2 mediated stress fiber formation and enhanced transmigration. However, because there is no commercially available antibody to detect Rock1’s phosphorylated Y722, investigating Rock1’s Y722 phosphorylation presents an extra challenge. Nonetheless, Rock1’s role in *Tg*WIP-Shp1/2 mediated modulation of DC motility warrants investigation in future studies.

Our analysis of Rock2’s phosphorylated Y722 using Western blot analysis demonstrated decreased phosphorylation Rock levels in DCs infected with *Tg*WIP^WT^ compared to DCs infected *Tg*WIP^Y150A/Y199A^ parasites (Fig. 6A). Notably, multiple bands were observed in uninfected DCs, whereas in infected DCs only Rock’s predicted band (160kDA) was observed. The analysis of pRock exclusively focused on the top Western blot bands, corresponding to Rock’s predicted bands. Rock cleavage in cells has been shown to be mediated by granzyme B and caspases [48, 49]. It is plausible that *Toxoplasma* infection inhibits the activity of proteins responsible for cleaving Rock, independently of *Tg*WIP. This is supported by studies indicating that *Toxoplasma* infection inhibits activation of caspase 3, leading to apoptosis inhibition [50]. Consequently, the absence of lower bands in infected DCs may be attributed to this phenotype.

It is known that cell migration also involves the dynamic interplay between the nucleus and the actin cytoskeleton [51]. Stress fibers are assembled under the nucleus of migrating cells which is mediated by myosin II [52]. F-actin stress fibers and actomyosin contraction forces subject the nucleus to tensional forces, which change the shape of the nucleus to allow for efficient cell migration [53]. Hence, the stress fiber formation and motility modulation of infected DCs may induce the nuclear morphological changes orchestrated by *Tg*WIP-Shp1/2 interactions.

*Toxoplasma* not only modulates the migratory properties of DCs but also those of various other leukocytes. For example, Drewry et al. demonstrated that *Toxoplasma* also hijacks the migratory properties of monocytes and macrophages, leading to increased motility on endothelial cells and enhanced migration through 3D tissues [54]. This same study found that parasitized monocytes, when treated with Rhosin (a Rho GTPase inhibitor) or Y-27,632 (a Rock inhibitor), traveled significantly decreased distances within collagen matrices. Although the *Toxoplasma*-secreted effector ROP17 has been implicated in activating the Rho-Rock signaling pathway [54], no direct *Toxoplasma*-mediated interaction with Rock that increases cell migration has yet been identified. It is likely that a similar Rho-Rock mediated mechanism for enhanced migration observed in human monocytes and murine macrophages might also exist in murine DCs. This is supported by our data showing that *Tg*WIP binds to Shp1/2 in human DCs (THP-1 derived DCs) in a manner akin to that in murine DCs (Fig. S2). However, whether *Tg*WIP is modulating the actin cytoskeleton and motility of monocytes and macrophages in a manner similar to DCs remains unexplored. Additionally, infected microglia exhibit enhanced transmigration in vitro and morphological changes, including loss of podosomes [55]. The *Toxoplasma-*derived *Tg*14-3-3 protein has the capability to induce hypermotility in both DCs and microglia [56]. Whether *Tg*WIP can also mediate these phenotypes in infected microglia is yet to be established. Furthermore, the role of *Tg*WIP-Shp1/2 interactions in promoting *Toxoplasma* dissemination in vivo warrants further investigation.

The focal adhesion kinase (FAK), a non-receptor tyrosine kinase, is another key molecule involved in cell migration and in regulating the actin cytoskeleton [57]. FAK is a highly tyrosine phosphorylated protein that localizes to focal adhesions, which are integrin enriched sites for cell adhesion. Focal adhesions are generated at the junctions between the ECM and integrin junctions during cell adhesion, spreading and migration. FAK’s Y576 and Y577 are phosphorylated within the kinase domain and promote FAK activation [58]. Studies have shown that the phosphorylation levels of FAK Y397 and Y576/577 are elevated after the knockdown and inhibition of Shp1 in mice, consequently activating FAK and the downstream focal adhesion pathway needed for cell migration [41]. Thus, another potential signaling pathway that could be mediated by *Tg*WIP-Shp1/2 interactions to modulate DC actin cytoskeleton after *Toxoplasma* infection is the Shp1-FAK signaling. However, this has yet to be investigated.

In addition to their roles in regulating cell motility and the actin cytoskeleton, Shp1 and Shp2 are important regulators of other cellular functions such as immune cell activity, tumor suppression, cell development, and cell proliferation [59–62]. *Leishmania major* hijacks Shp1 to inhibit cross presentation in DCs, thereby evading CD8 T cell responses [63]. Shp2 has been implicated in decreasing the expression of TNFα and IFNγ in natural killer (NK) cells after *Toxoplasma* infection [64]. Our study focused mainly on the role of Shp1 and Shp2 on DCs’ motility and migration that could contribute to the *Tg*WIP-mediated ‘Trojan Horse’ dissemination of *Toxoplasma*.

Given the broad range of Shp1 and Shp2 functions, it is important to investigate whether interactions between *Tg*WIP and Shp1/2 influence *Toxoplasma* pathogenesis by downregulating immune functions in infected DCs, such as antigen presentation or cytokine secretion, thereby facilitating immune evasion. Although previously we reported that in vivo competition between wild-type and ∆*tgwip Toxoplasma* at equal ratios intraperitoneally resulted in increased numbers of ∆*tgwip* parasites in the peritoneum seven days post infection. This suggests that ∆*tgwip* parasites are not more susceptible to the host immune response, but rather have a significant impairment in disseminating out of the peritoneum.

Our data suggest that the interactions of *Tg*WIP with Shp1/2 predominantly contribute to the stress fiber-mediated hypermotility phenotype, rather than the dissolution of podosomes. This points to a distinct *Tg*WIP-mediated mechanism underlying the dissolution of podosomes. Given the established roles of the WAVE complex in generating cell protrusions such as lamellipodia and phagocytosis, alongside the involvement of the WASP-Nck1/Grb2 pathway in the formation of filopodia and podosomes, it is likely that the dissolution of podosomes is associated with *Tg*WIP’s interaction with the WAVE complex and/or Nck1/Grb2. The precise nature of *Tg*WIP’s interaction with the WAVE complex and Nck1/Grb2, and its influence on podosome dissolution in infected DCs, remains an area for further investigation.

### Electronic supplementary material

Below is the link to the electronic supplementary material.

Supplementary Material 1

## Data Availability

All the data are contained within the article and supporting information. Parasite strains in this paper will be available upon request.
