# Supplementary material for: The Toxoplasma secreted effector TgWIP modulates dendritic cell motility by activating host tyrosine phosphatases Shp1 and Shp2
Source: Cell Mol Life Sci. 2024 Jul 9;81(1):294. doi: 10.1007/s00018-024-05283-3 (PMC11335217; doi:10.1007/s00018-024-05283-3)
Supplement: Supplementary file 1 — Supplementary Material 1 [file 18_2024_5283_MOESM1_ESM.docx]

**Supplemental figures**


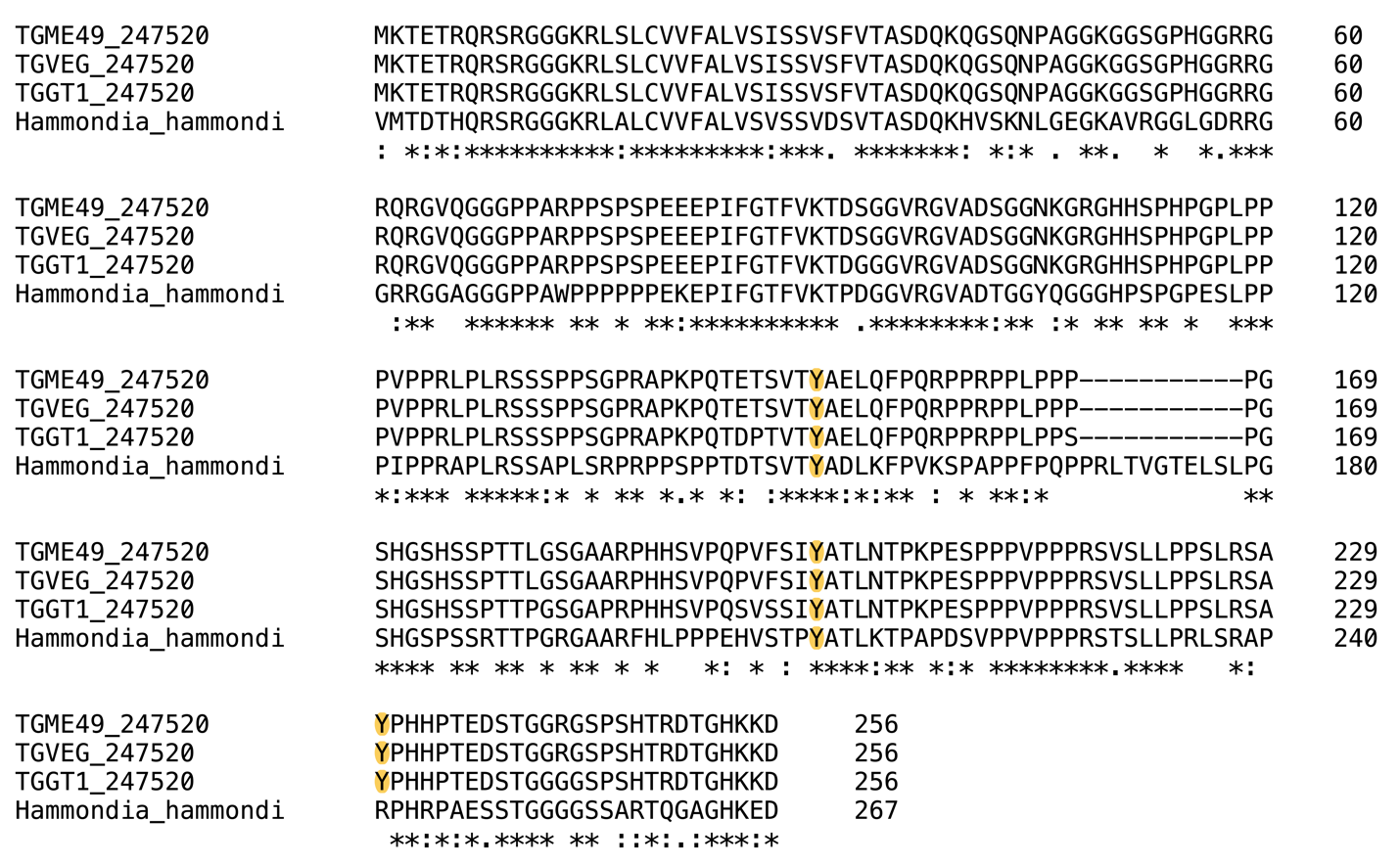


**Fig. S1** *Tg*WIP amino acid sequence (ToxoDB gene ID 247520) alignment of *Toxoplasma* strains ME49, VEG, GT1, and *Hammondia hammondi*. Tyrosine’s position 150 and 199 is conserved in *Hammondia*, whereas the 230 tyrosine is not.


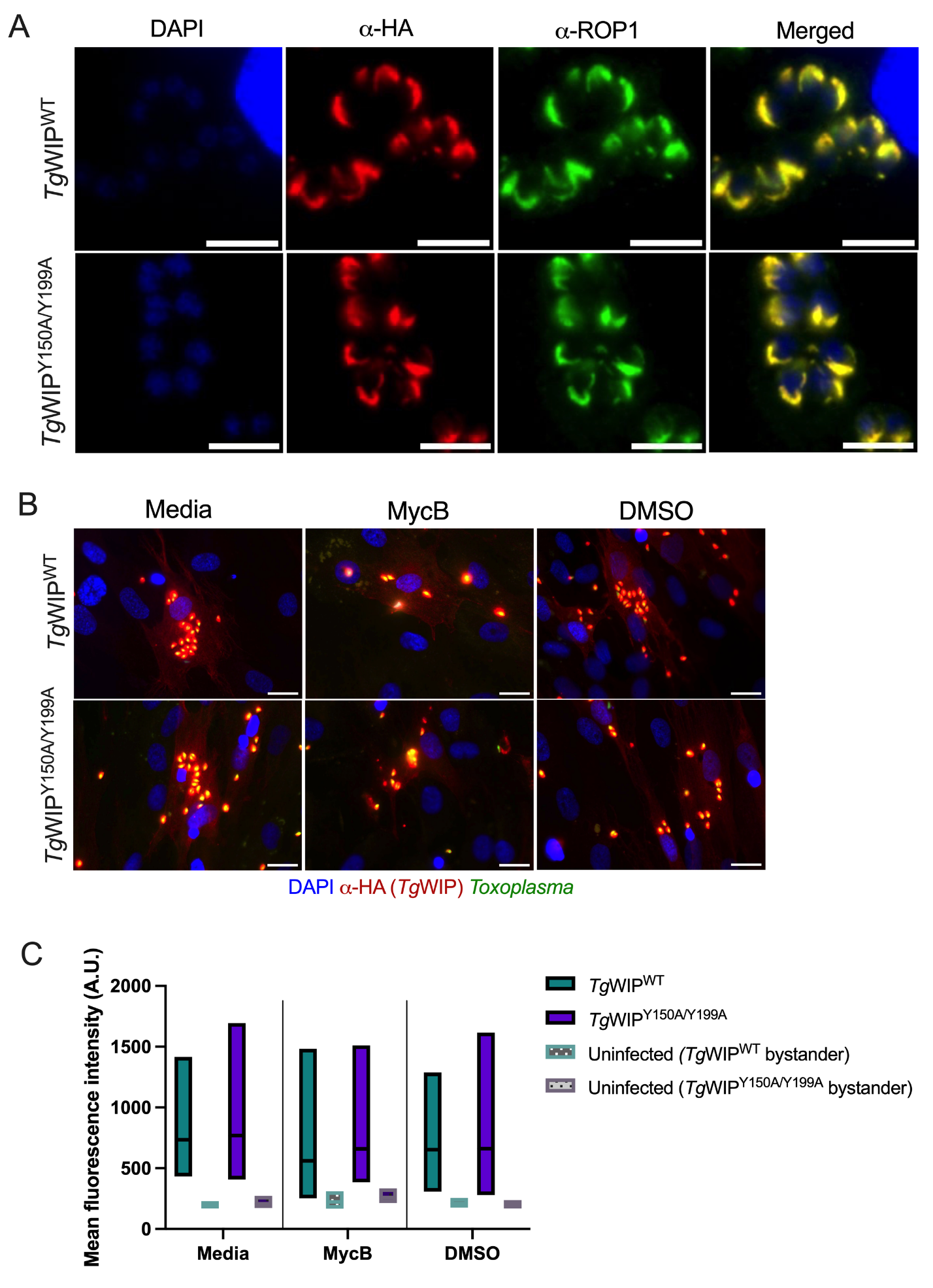


**Fig. S2 A** *Tg*WIP and rhoptry organelle colocalization of *Tg*WIP^WT^ or *Tg*WIP^Y150A/Y199A^ parasites. Immunofluorescence assay on HFFs infected with *Toxoplasma* containing either endogenously HA-tagged wildtype *Tg*WIP (*Tg*WIP^WT^) or *Tg*WIP expressing 150 and 199 tyrosine to alanine mutations (*Tg*WIP^Y150A/Y199A^). ROP1 was used as a marker for rhoptry organelles. **B** *Tg*WIP^WT^ or *Tg*WIP^Y150/Y199A^ *Toxoplasma* were treated with 100 μM mycalolide B (mycB), an irreversible inhibitor of actin polymerization [[65]](https://paperpile.com/c/DcjJSh/hPYNt), parasites treated with MycB are unable to invade into the host cell but can still attach to host cells and discharge rhoptry contents. MycB, DMSO-treated, or parasites left in media were added to HFFs for 3 h. Immunofluorescence images shown of mock (media), mycB, or DMSO treated *Toxoplasma* stained for HA. Scale bar measures 10 μm. **C** The mean HA fluorescence intensity of uninfected (bystander) HFFs or *Toxoplasma* infected HFFs treated with media, mycB, or DMSO was determined 3 h after infection.

**
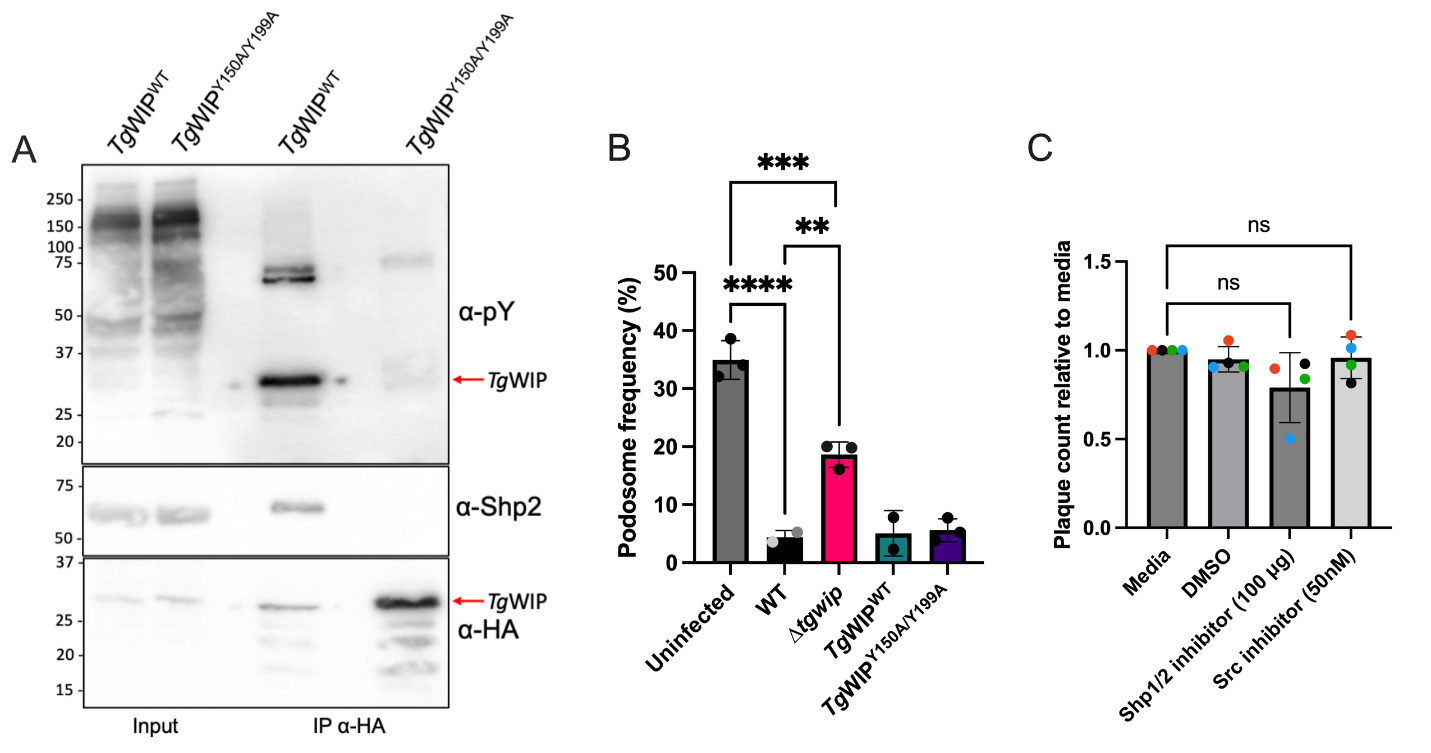
**

**Fig. S3 A** Human THP-1 derived DCs were infected with *Tg*WIP^WT^ or *Tg*WIP^Y150A/Y199A^ parasites. Shown is the Western blot using pY, Shp2, and HA antibodies on total lysate or immunoprecipitated *Tg*WIP. **B** Quantification of the percentage of THP-1 DCs containing podosomes infected with corresponding *Toxoplasma* strains, 4 h post infection**.** **C** Plaque assay of WT ME49 *Toxoplasma* incubated with Shp1/2 inhibitor (NSC-87877, 100 μg), Src inhibitor (Dasatinib, 50nM), or solvent control (DMSO). Bar graph shows the average number of plaques counted at day 6, from at least 3 independent experiments relative to the number of plaques of *Toxoplasma* in media only. ns, non-significant difference; two-way ANOVA, Dunnett's multiple comparisons test.


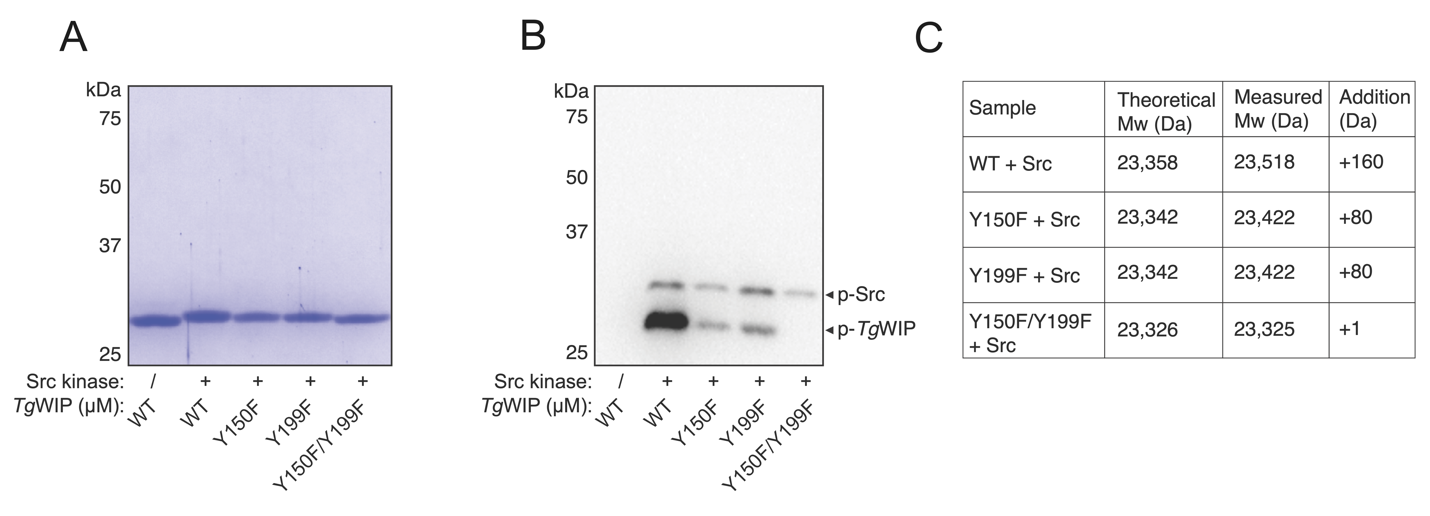


**Fig. S4 A** Coomassie blue-stained SDS-PAGE gel showing *in vitro* phosphorylation of *Tg*WIP via Src kinase and high purity and consistency of protein added to each reaction. **B** Western blot after *in vitro* phosphorylation via Src kinase revealed only Y150 and Y199 residues of *Tg*WIP were phosphorylated. **C** Table summarizing mass spectrometry results measuring intact molecular weight of *Tg*WIP samples shown in A) and B), confirming that only Y150 and Y199 residues could be phosphorylated by Src kinase.

**
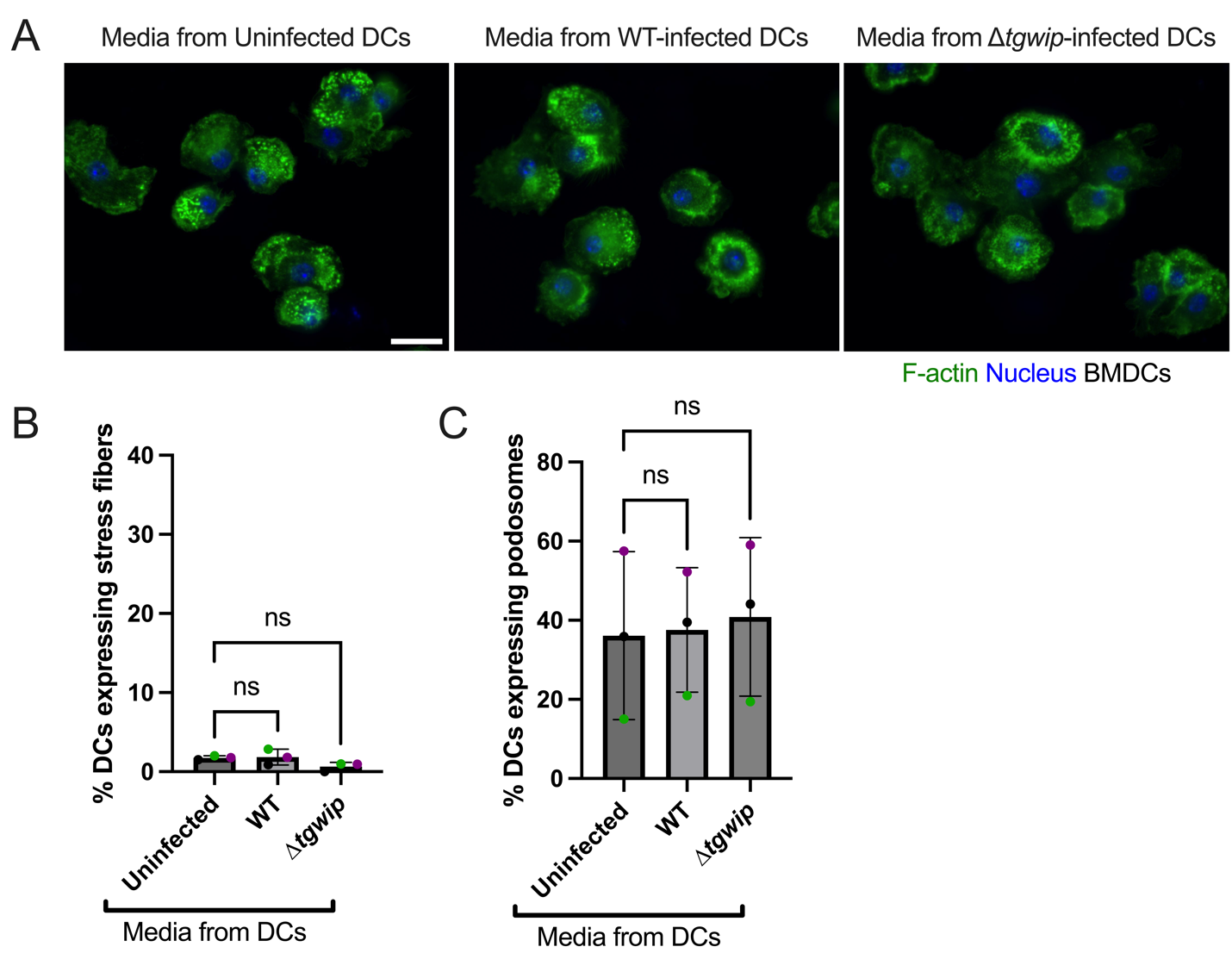
**

**Fig. S5** Media from infected BMDCs has no effect on stress fiber and podosome expression of uninfected BMDCs. Uninfected BMDCs, or BMDCs infected with either WT or ∆*tgwip Toxoplasma* were infected for 4 h at MOI 3. BMDCs were then spun down and the supernatant (media) was collected and transferred to uninfected BMDCs and incubated at 37°C for 16 h. **A** Immunofluorescence images of uninfected BMDCs incubated with the media of either uninfected or infected BMDCs, stained for F-actin and DAPI. Bar graphs shows the average percent of at least 100 uninfected BMDCs expressing **B** stress fibers or **C** podosomes, from 3 independent experiments. Scale bar measures 20 μm. ns, non-significant difference; two-way ANOVA, Dunnett's multiple comparisons test.


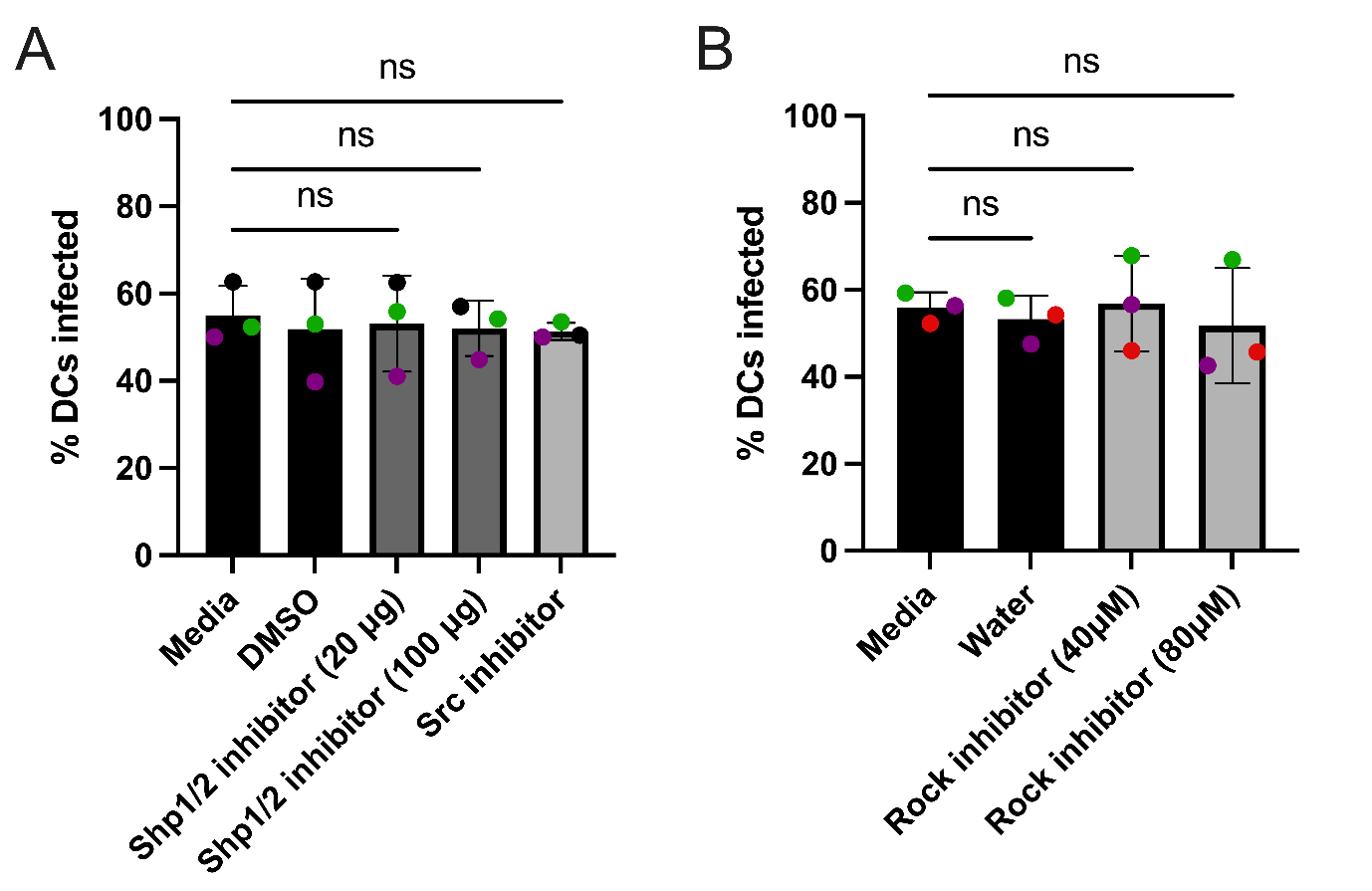


**Fig. S6** No effect of Shp1/2, Src, and Rock inhibitor on DC infection rate. Bar graphs showing percent of DCs infected with WT ME49 *Toxoplasma* in the presence of **A** Shp1/2 inhibitors (at the indicated concentrations), Src inhibitor (50nM), and **B** Rock inhibitor (at the indicated concentrations), solvent control, or left in media only. Data from at least 3 independent experiments. ns, non-significant difference; two-way ANOVA, Dunnett's multiple comparisons test.

**
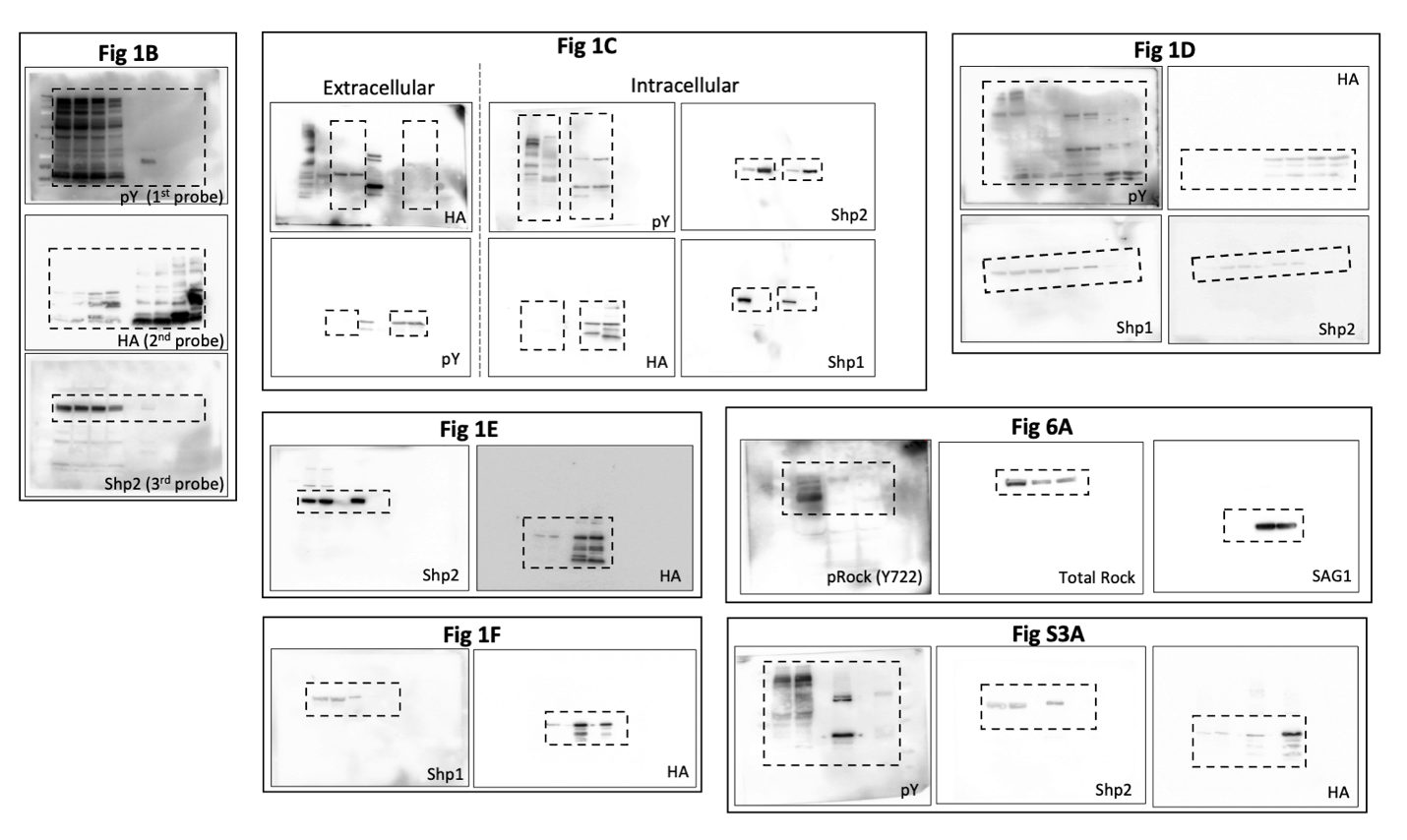
**

**Fig. S7 All uncropped Western blot images in this study.**
